# High-resolution in vivo kinematic tracking with customized injectable fluorescent nanoparticles

**DOI:** 10.1126/sciadv.adu9136

**Published:** 2025-10-01

**Authors:** Emine Zeynep Ulutas, Amartya Pradhan, Dorothy Koveal, Jeffrey E. Markowitz

**Affiliations:** ^1^ Wallace H. Coulter Department of Biomedical Engineering, Georgia Institute of Technology and Emory University, Atlanta, GA, USA.; ^2^ Neuroscience Graduate Program, Emory University, Atlanta, GA, USA.

## Abstract

Markerless keypoint trackers, algorithms trained to identify specific points on an animal, have transformed neuroscience and enabled movement quantification without the attachment of surface markers. However, while these approaches represent a major advancement, they have yet to achieve the precision of motion capture in humans and have not been benchmarked against ground-truth datasets in common model organisms*.* Moreover, the extent to which they can be used to reliably track movement kinematics remains unclear. Here, we describe a tracking method that uses near-infrared quantum dots as injectable markers. We demonstrate that our tags can be injected beneath the skin or into joints and imaged for months in freely moving mice noninvasively using standard cameras. Last, we create a large ground-truth dataset for training the next generation of markerless systems. By using injectable landmarks in the joints, this method brings us closer to understanding how the brain controls skeletal movements.

## Teaser

Injectable fluorescent nanoparticles were used to track positions on and inside of freely moving animals at high resolution.

## INTRODUCTION

Animal movement has traditionally been characterized by manually scoring video footage or direct observation (*1*–*4*). Recent technological advances in machine learning have enabled the study of unrestrained, naturalistic movements with unprecedented speed and accuracy, especially in the field of markerless keypoint tracking, which has been transformative for neuroscience (*5*–*10*). State-of-the-art methods such as transfer learning with convolutional networks (*9*), U-Net–inspired architectures (*10*, *11*), and three-dimensional (3D) convolution networks (*6*) have enabled tracking of discrete computationally defined points on the surface of laboratory animals trained on a small number of hand-labeled video frames. These advances, in turn, have enabled new methods for classifying animal behaviors (*12*–*14*) and quantifying movement (*15*, *16*).

However, because of variations in experimental setups across laboratories, markerless keypoint trackers still require hand-labeling datasets. This can introduce jitter due to variation between annotators and the inherent ambiguity of labeling certain body parts based solely on surface features, such as positions along the back of a mouse. In rats, where markerless keypoint trackers have been systematically compared against skin-attached fiducials, their predictions were estimated to have precision on the order of ±10 mm (*17*), comparable to the distance between many key landmarks on the mouse (*18*). This contrasts with commercial motion capture systems used with humans, which have a demonstrated precision of ~±0.1 mm (*19*–*21*).

Another limitation of markerless keypoint trackers is that they are trained to identify points on the outside of the animal’s body using surface features visible in videography. As joint and skeletal kinematics can be obscured by soft tissue and fur in rodents (*22*, *23*) and humans (*24*, *25*), it remains unclear whether movement of the skeleton can be resolved in this way. This is important because the brain directly controls the muscles, which exert complex forces on the skeleton. The joints exert important constraints on skeletal motion and thus shape resulting animal movements (*26*). It remains unclear whether tracking motions on the surface of the skin is a viable strategy for resolving the brain’s control of movement and how it breaks down in disease and injury (*22*, *25*, *27*–*29*).

Although technically demanding, it is possible to directly observe the skeletal system in live rats using x-ray videography, which has demonstrated the principle that skin-derived joint angles and kinematics can greatly diverge from those derived directly from the skeleton (*22*). While this technique has very high spatial resolution, x-ray videography is challenging to set up in individual laboratories and can be limited by radiation dosage, allowing only brief imaging sessions (*30*–*32*). Imaging over long periods of time is important for longitudinal experiments, such as observing the development of movement or disease progression.

An alternate strategy would be to implant optical tags in key landmarks inside the body, including the joints, that can be measured noninvasively. To be successful, this approach must satisfy two important criteria. First, the implanted optical tag must be detectable from outside of the animal. Prior studies suggest that near-infrared I (NIR-I; 650 to 900 nm) is an ideal spectral window for noninvasive imaging due to minimal light absorption and scattering by skin (*33*–*35*) and hemoglobin (*36*). Quantum dots (QDs) are one of the few fluorescent materials in this spectral range that are photostable (i.e., minimal photobleaching) and bright (i.e., high quantum yields and extinction coefficients), making them an attractive material relative to NIR-fluorescent proteins and dyes (*34*, *37*–*39*). Second, the implanted optical tag should be long-lasting to support long-term studies of movement in an animal. In addition to being photostable, it is therefore important for the tag to be biocompatible and to have a long half-life in vivo. QDs are widely used in the life sciences and thus are readily available in numerous biocompatible formulations (*39*–*42*) and have been used in vivo (*43*, *44*). However, the in vivo half-life of different QD formulations has not previously been investigated.

Here, we present a tracking method called QD-Pi (quantum dot-based pose estimation in vivo). QD-Pi uses NIR-I–emitting QDs as injectable, bright, long-lived optical probes that can be imaged noninvasively in freely moving animals using standard machine-vision cameras. We demonstrate that QD-Pi can be used to reliably track keypoint markers in the joints while mice freely move in an open plexiglass arena. First, we establish that QDs can be imaged through the skin by injecting QDs subdermally in the subcutaneous fatty tissue. Second, using subdermal injections, we identify specific QD formulations that serve as bright, long-lived physical fiducial markers. This includes antibody-conjugated QDs that can be targeted to ultralong-lived proteins like collagen to further enhance signal longevity in vivo up to 4 months postinjection. This phase of testing also presented an opportunity to collect a large dataset for ground-truthing markerless keypoint trackers, wherein subdermal QDs and body surface reflectance were imaged simultaneously. We used this dataset to benchmark the performance of an existing markerless keypoint tracker, Social LEAP Estimates Animal Poses (SLEAP) (*10*), relative to QD fiducials embedded in the skin. We found that QDs can be used to enhance the accuracy of markerless keypoint trackers. Last, we demonstrate that QDs can be injected intra-articularly for directly tracking joint kinematics. The joints impose constraints on skeletal movement, and imaging joint motion in 3D is an important step toward the overarching goal of directly measuring skeletal kinematics. Ultimately, QD-Pi addresses a critical need in the study of motor control in neuroscience and sets the stage for a generalizable method to track movement and skeletal kinematics in freely moving animals.

## RESULTS

### QDs are viable injectable optical tags in mice

We developed a protocol to inject QDs subdermally into mice to establish that QDs can be imaged through the skin while mice freely move in an arena. We selected QDs as a fluorescent marker because of their brightness, photostability, and broad emission spectral profiles within the NIR-I (650 to 900 nm) range, which is an ideal window for imaging through skin (*33*–*35*) and blood (*36*). QDs are also biocompatible, and their surface chemistry can be readily modified with a variety of biologically functional surface coatings. Here, we used QDs with a fluorescence emission peak at 800 nm (referred to as QD800 throughout) coated in polyethylene glycol (PEG), previously demonstrated to be biocompatible when introduced into animals (*45*).

**Fig. 1.**
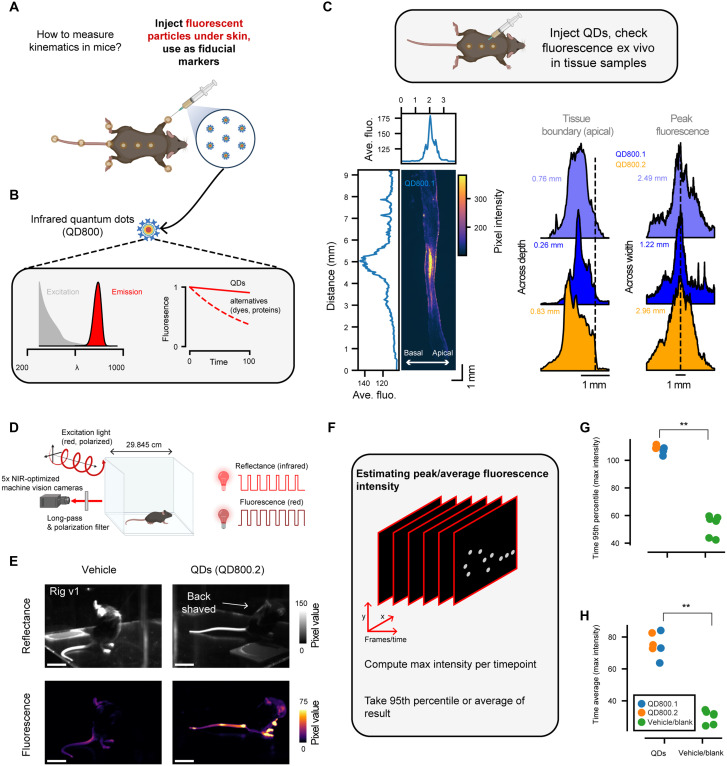
QDs can act as optical markers when introduced into the skin. (**A**) Schematic representation of the QD injection procedure. (**B**) Schematic representation of the basic optical properties of NIR-emitting QDs. (**C**) Histological examination of QDs injected into the back of a mouse. Top: Schematic of the experiment. Injectable QDs were introduced into the skin, after which tissue samples were harvested for imaging. Bottom left: Example fluorescence image of a skin sample after QD injection (QD800.2). Mean projections are shown along the *x* and *y* axes. Bottom right: Fluorescence probability densities at different tissue depths aligned to the tissue boundary (left) or the fluorescence peak (right). Each plot corresponds to a separate injection (QD800.1 or QD800.2). Full width at half maximum is shown next to each distribution. (**D**) Schematic of the plexiglass arena and optical configuration for in vivo imaging. Reflectance and fluorescence images are collected near-simultaneously using IR-emitting LEDs and polarized NIR-I-emitting LEDs, respectively. Five machine vision cameras equipped with long-pass and polarization filters are used to collect images. (**E**) Example reflectance and fluorescence images from vehicle (left) and QD-injected mice (QD800.2, right). Scale bars, 50 pixels. (**F**) Schematic illustrating how QD fluorescence is measured. First, the max intensity is computed per frame, and then either the 95th percentile or the mean is computed across all frames for each mouse and camera. (**G**) The 95th percentile pixel intensity comparison of vehicle/blank (*n* = 6 mice) and QD mice (*n* = 3 QD800.1 and *n* = 3 QD800.2 mice) (*P* = 0.002, *U* = 36, *f* = 0, Mann-Whitney *U* test). Measurements were averaged across the five cameras. (**H**) Average pixel intensity comparison between vehicle/blank or QD injection (*P* = 0.002, *U* = 36, *f* = 0, Mann-Whitney *U* test).

We first tested whether QDs could form discrete “tags” without dispersing through the skin and underlying tissue (Fig. 1, A to C). Ideally, the resulting tag would be large enough to resolve on a standard machine vision camera yet small enough to mark many different points on the body. As an approximation, we assumed as a basic criterion that fluorescence points should be at least 1 mm in diameter to enable high-resolution tracking (roughly 5 pixels assuming an object distance of 12 inches, or 30.48 cm, to the camera based on our camera calibrations).

To accomplish this, we developed a simple microinjection protocol whereby small quantities (<5 μl) of QD800 particles could be injected under the skin of a briefly anesthetized live mouse using a glass micropipette (see Materials and Methods). We selected two QD800 formulations. Vascular labels, a QD suspension originally designed for visualization of the vasculature (referred to here as QD800.1), and cell labels, a QD suspension of nanoparticles functionalized with a peptide that facilitates its uptake into cells (referred to here as QD800.2) for use in initial tests based on their prior use in vivo and desirable spectral properties. We then identified three subdermal locations along the back (dorsal midline) for injection and confirmed the size and depth of the fluorescence spots in extracted ex vivo tissue sections (Fig. 1C). Histology revealed that injections of QD800.1 or QD800. 2 yielded a bright fluorescent spot in adipose tissue just beneath the dermis, at a depth of 0.26 to 0.83 mm beneath the skin with a spot full width at half maximum of 1.22 to 2.96 mm (approximately 6 to 15 pixels in images acquired with machine vision cameras equipped with standard 8-mm focal length lenses at a distance of 12 inches (30.48 cm), the approximate distance from each camera to the center of the arena in our setup). We then extended our method to 14 subdermal locations: dorsal and ventral injections to each paw, three injections along the tail, and three injections along the spine (Fig. 1A). It is important to note that injection areas that involved fur required shaving and treating with hair removal cream so that fur did not interfere with imaging.

To image QD800.1 or QD800.2 in freely moving mice, we constructed a plexiglass imaging arena surrounded by five NIR-optimized machine vision cameras, which was an open-top cube with an edge length of 29.845 cm (Fig. 1D and fig. S1). The cameras were equipped to collect both reflectance images to visualize the location of the mouse and fluorescence images to visualize QD800 fluorescence (730-nm excitation, >800-nm emission; we refer to this lighting setup as Rig v1). To explicitly estimate the contribution of non-QD–dependent signals in the fluorescence channel, a separate group of animals received either vehicle or no injection (see Materials and Methods). Following optical tag embedding, mice were introduced into the plexiglass arena and were imaged during free behavior over 3 to 5-min-long sessions (Fig. 1E and movie S1). Relative to vehicle-injected mice, mice injected with QD800.1 or QD800.2 exhibited clearly resolvable fluorescence [signal-to-noise ratio (SNR) ~ 2.51 ± 0.25 SD, where we define the SNR as average fluorescence across the whole image per QD-injected mouse relative to average blank/vehicle-injected mice, *n* = 6 QD800-injected mice, composed of *n* = 3 QD800.1 and *n* = 3 QD800.2 mice, and *n* = 6 blank/vehicle-injected mice; Fig. 1, F to H, and fig. S2].

### As free nanoparticles, QD signal decays within hours in vivo

**Fig. 2.**
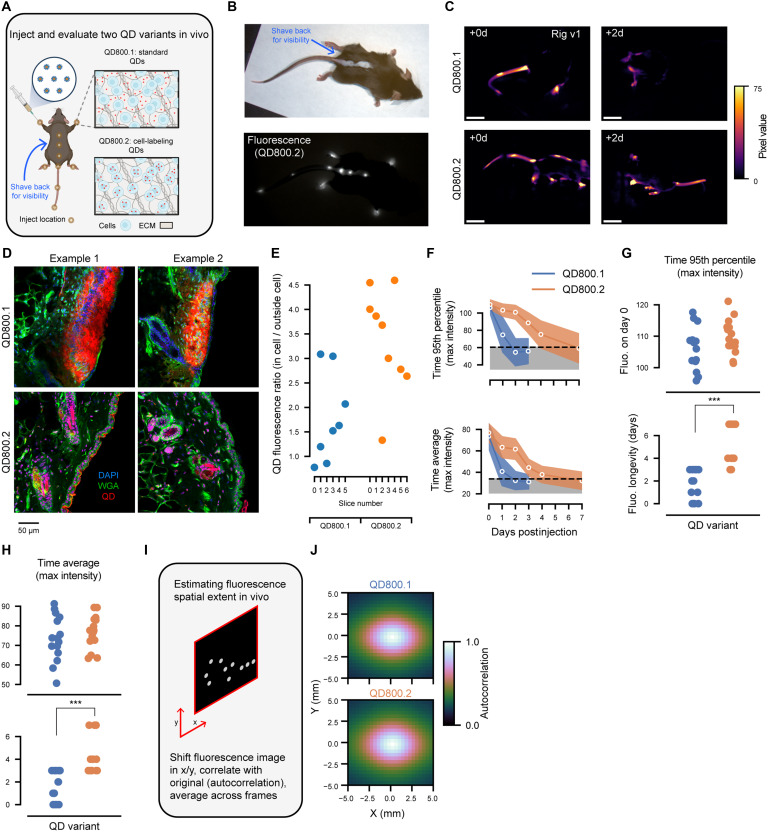
Half-life of QDs in buffer (QD800.1) and QDs that enter cells (QD800.2). (**A**) Schematic of hypothesized QD800.1 and QD800.2 localization. (**B**) Reflectance and fluorescence image of a mouse immediately after injection of QD800.2. (**C**) Example fluorescence images from QD800.1 (top) and QD800.2 (bottom) imaged at 0 and 2 days postinjection. Scale bars, 50 pixels. (**D**) Confocal images from injections into the backs of mice with either QD800.1 (top) or QD800.2 (bottom). Tissue was stained with DAPI (for nuclei) and wheat germ agglutinin (WGA; for cell membranes). (**E**) Ratio of QD fluorescence inside of cells to outside of cells (*P* = 0.015, *f* = 0.85, Mann-Whitney *U* test; *n* = 9 fields of view from *n* = 2 tissue samples/injections for QD800.2 and *n* = 8 fields of view from *n* = 2 tissue samples/injections for QD800.1). (**F**) Either 95th percentile (top) or average pixel intensity (bottom) plotted as a function of days postinjection for both variants (see Fig. 1F). Shown is the mean (line) and one SD (shaded region) across each quantity for every mouse/camera view pair (*n* = 15). Gray region indicates 99th percentile confidence interval for vehicle/blank mice. Same conventions used throughout. (**G**) Initial brightness (top) and the fluorescence longevity (time for the trace for each mouse/camera pair to cross below the 99th percentile of vehicle/blank mice, bottom) for both variants calculated from 95th percentile pixel intensity over time (*P* = 0.23, *U* = 142, *f* = 0.63 for brightness; *P* = 6.9 × 10^−6^, *U* = 219, *f* = 0.97 for longevity, Mann-Whitney *U* test; *n* = 15 mouse/camera pairs each). (**H**) Same as Fig. 2G, except computed using average pixel intensity over time (*P* = 0.41, *U* = 133, *f* = 0.59 for brightness; *P* = 7.5 × 10^−5^, *U* = 204, *f* = 0.91, for longevity, Mann-Whitney *U* test; *n* = 15 mouse/camera pairs each). (**I**) Schematic of spatial autocorrelation calculation to measure the length-scale of QD-induced fluorescence. (**J**) Average in vivo spatial correlation of fluorescence across all mice and camera views for both QD800.1 (top) and QD800.2 (bottom). d, days.

For future applications of our method in standard neuroscience experiments (e.g., photometry, imaging, pharmacological, or optogenetic manipulations) or for longitudinal tracking of an animal’s movements, our optical tags should retain fluorescence for as long as possible—ideally on the order of weeks. To test the longevity of QD800 fluorescence, we monitored fluorescence in freely moving mice across multiple days after subdermal injection (Fig. 2, A to C). As QD800.1 is a suspension of nanoparticles (10 to 20 nm in diameter) without targeting tags, we speculated that they would quickly disperse between cells within a tissue. On the other hand, QD800.2 is a suspension of nanoparticles functionalized with a peptide that facilitates its uptake into cells (Fig. 2, A and B), which we speculated would lead to enhanced fluorescence longevity in vivo.

To visualize the dynamics of these QDs in the skin, we performed QD800.1 and QD800.2 injections along the back of an additional cohort of animals and harvested the tissue for histology (*n* = 4 tissue samples/injections, *n* = 2 with QD800.1, and *n* = 2 with QD800.2; see Materials and Methods). We stained the cell membranes and nuclei and visualized the fluorescence with confocal microscopy in multiple fields of view to confirm whether the QDs were located inside or outside of the cells (Fig. 2D). We then quantified the degree to which QDs entered the cells as the ratio of the average QD fluorescence inside compared to outside of cells. We found that the median ratio (intracellular/extracellular) for QD800.2 (3.7) was more than twofold greater than the ratio for QD800.1 (1.6) (Fig. 2E).

Longitudinal in vivo imaging of mice injected with QD800.1 and QD800.2 revealed that signal decayed within 1.6 ± 1.4 days and 5.3 ± 1.7 days, respectively (mean ± SD; Fig. 2, F to H; see figs. S3 and S4 for raw data). This supported our hypothesis that cell internalization could prolong visualization of the optical tag. Since QD800.1 and QD800.2 both use the same underlying fluorescent nanoparticle, it is also suggested that the decay in fluorescence was not due to photobleaching. Average spatial autocorrelation of the fluorescence images across all animals and camera views showed that both QD800 variants remained localized to the injection site in vivo immediately following injection (Fig. 2, I and J).

### Microbead-immobilized QDs yield signal that can be imaged for weeks

**Fig. 3.**
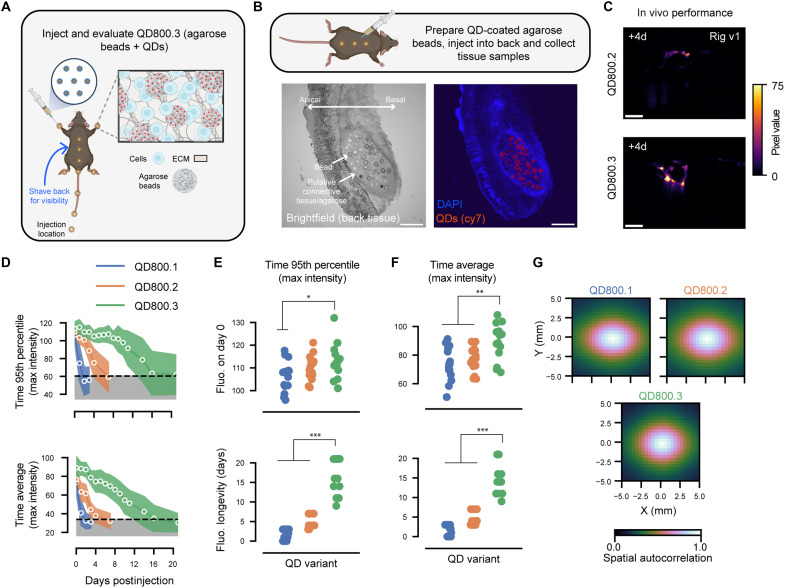
Custom agarose bead QD mixture (QD800.3) outperforms all other variants in vivo. (**A**) Schematic representation of QD injection sites with QD800.3. ECM, extracellular matrix. (**B**) Brightfield (left) and fluorescence (right) images from the same tissue sample taken from the back 1 day after QD800.3 injection. Scale bars, 500 μm. (**C**) Example fluorescence images from QD800.2 (top) and QD800.3 (bottom) imaged at 4 days postinjection. Scale bars, 50 pixels. (**D**) Either 95th percentile (top) or average (bottom) pixel intensities plotted as a function of days postinjection for all three variants. Line indicates the average and shaded region one SD across mouse/camera pairs. (**E**) Initial brightness (top) and the decay rate (bottom) for all variants computed using the 95th percentile across time (*P* = 0.013/0.19, *U* = 173/144.5, *f* = 0.77/0.64 for brightness; *P* = 2.5 × 10^−6^/2.3 × 10^−6^, *U* = 225/225, *f* = 1/1 for longevity, Mann-Whitney *U* test; *n* = 15 mouse/camera pairs each, QD800.3 compared with QD800.1/QD800.2). (**F**) Same as Fig. 3E, except computed using the average across time (*P* = 0.004/0.009, *U* = 182/176, *f* = 0.81/0.78 for brightness; *P* = 2.7 × 10^−6^/2.5 × 10^−6^, *U* = 225/225, *f* = 1/1 for longevity, Mann-Whitney *U* test; *n* = 15 mouse/camera pairs each, QD800.3 compared with QD800.1/QD800.2). (**G**) Spatial autocorrelation for all three variants in vivo.

Although QD800.2 outlasted QD800.1, each was only detectable for days, not weeks. We hypothesized that immobilizing QDs on large microparticles would further prolong the signal of the optical tag by minimizing diffusion and discouraging clearance from the body. Prior work has shown that >30-μm-diameter microbeads injected subdermally in humans can form long-lasting deposits that persist for multiple years (*46*). We therefore tested our hypothesis by combining biotin-functionalized QDs with streptavidin-functionalized microbeads (QD800.3; fig. S5, A to F) and injecting them subdermally as before. We tested six commercially available microbeads and found that porous ~85-μm-diameter high-capacity agarose beads yielded bright beads (fig. S5A). We confirmed that QD800.3 formed dense deposits in the skin that persisted in the animal (Fig. 3, A, B, and G, and fig. S6), increasing the imaging window to >16 days (16.6 ± 4.6 days; QD800.3, *n* = 3 mice; Fig. 3, C to F). This longevity is critical to tracking the progression of movement disorders in certain mouse disease models, such as models for Parkinson’s disease (*47*), and over the course of standard systems neuroscience experiments that include reading and writing neural activity over multiple sessions.

### Optimizing excitation wavelength and imaging optics maximizes SNR

**Fig. 4.**
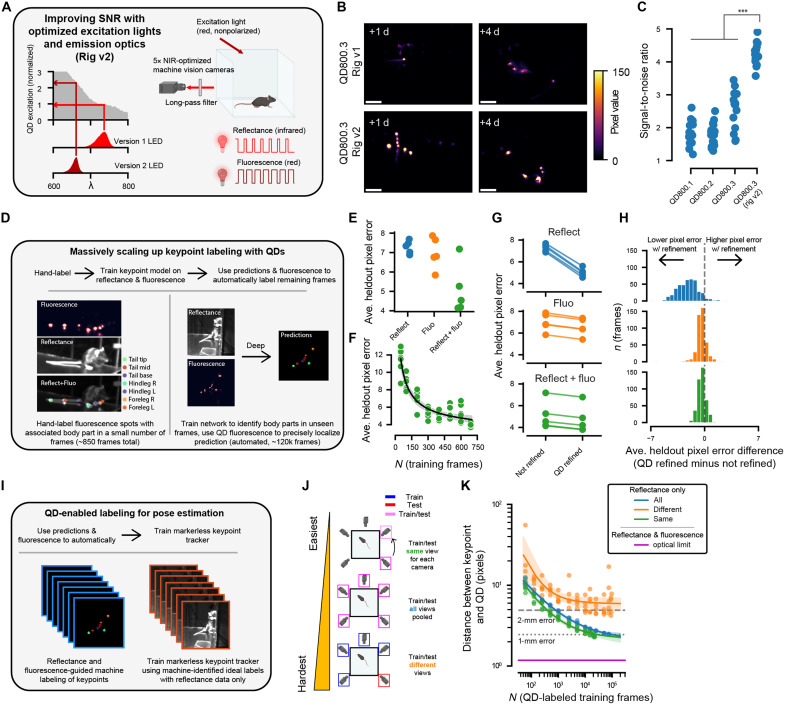
Combining QD800.3 and an optimized imaging rig enhances SNR and enables scaling of markerless keypoint trackers. (**A**) Schematic of changes. (**B**) Fluorescence images from mice injected with QD800.3 using Rig v1 (top) or Rig v2 (bottom). Scale bars, 50 pixels. (**C**) SNR across all variants and rigs, estimated by taking the SD across *x* and *y* axes for each fluorescence frame and then averaging the result across all frames for each mouse/camera view pair (QD800.3 Rig v2 comparison with all other variants, *P* = 3.4 × 10^−6^, *U* = 0, *f* = 1, Mann-Whitney *U* test, *n* = 15 mouse/camera pairs). The noise level was estimated by calculating the same metric for blank/vehicle mice. (**D**) Schematic illustrating scaling up of labeling (see Materials and Methods). L, left; R, right. (**E**) Keypoint prediction localization error relative to hand-labeled points using reflectance images (“reflectance only”), fluorescence images (“fluorescence only”), or their combination (“both”). Errors are averaged over all keypoints and frames for a model trained with a new random seed (*n* = 5 restarts). (**F**) Performance of the both model after subsampling the training dataset. (**G**) Average held-out error for a model trained using a different random seed (*n* = 5 restarts) with and without QD fluorescence refinement (see Materials and Methods). (**H**) For each frame, the difference in pixel error is shown with and without fluorescence refinement. (**I**) Schematic for using machine-labeled frames to train markerless keypoint trackers on reflectance data only. (**J**) U-Nets were trained and tested using data from the same camera view (same, top), all camera views (all, middle), or trained on four camera views and tested on one heldout view (different, bottom). (**K**) Results from training U-Nets according to Fig. 4J. Error is the distance between the prediction and the nearest QD fluorescence peak. For model performance curves, lines reflect the median power-law decay fit, and shaded region indicates 95% bootstrap confidence interval.

Having confirmed the use of QDs as discrete, bright, long-lived optical tags, we next hypothesized that the SNR could be improved by optimizing the imaging hardware (camera, excitation lights, and optical filters). Specifically, we speculated that tuning the excitation and emission optics to better match the optical properties of QD800 without being appreciably visible to the mouse (wavelengths equal to or above 650 nm) could substantially improve fluorescence signal (*48*). Initial recordings were made using 730-nm light-emitting diodes (LEDs) with polarization filters for excitation, and cameras were equipped with 830-nm long-pass filters and polarization filters tuned to the orthogonal orientation to minimize stray light (Rig v1; Fig. 4A). While 730 nm is ideal for penetrating the skin, it only excites QD800 particles at 1% efficiency, and an 830-nm long-pass filter clips the peak emission (*35*). Hence, we altered our setup to use 660-nm LEDs, thereby doubling the excitation efficiency, and a 780-nm long-pass filter to maximize collection of the emitted light (Rig v2; Fig. 4A and fig. S7A). These changes increased SNR > twofold for mice injected with QDs subdermally as before (Fig. 4, B and C, and fig. S8), which provided unambiguous detection of keypoints with noise characteristics suitable for use in downstream applications (fig. S9), such as training markerless keypoint models. We call this final iteration of QD800.3 tags combined with the upgraded imaging system and analysis pipeline, QD-Pi.

### QD800 images can help profile and train markerless keypoint trackers

Having built QD-Pi, we set out to collect a unique, large-scale dataset that could be used to both train and benchmark markerless keypoint trackers. Specifically, we collected rapidly alternating reflectance and fluorescence images from freely moving mice injected with QD800.3 subdermally (*n* = 3 additional mice), which enabled the collection of high-quality video-rate data surface features of the mouse registered to ground-truth key points inside of the mouse (Fig. 4D). This system allowed us to test the accuracy of commonly used markerless keypoint tracking algorithms relative to ground-truth optical markers placed inside of the body. Here, we focused on the commonly used U-Net architecture available in the SLEAP package (*10*).

Our first goal was to automatically label the body part associated with each QD800.3 injection. To accomplish this, we hand-labeled 862 frames across the five camera views (see Materials and Methods). Fluorescence and reflectance frames were superimposed so that the labeler could identify both a given body part and peak QD800 fluorescence (Fig. 4D). These hand-labels were then used to train a U-Net using both reflectance and fluorescence data, to identify which QDs correspond to which body part (fig. S10). The resulting dataset comprised 114,629 frames of machine-labeled keypoints in three mice across five camera views, which we call QD-Pi-120K (see Materials and Methods). This presented us with an opportunity to test the scaling properties of common markerless keypoint trackers relative to ground-truth fiducial markers (Fig. 4, E to H). We speculated that such a large dataset would enable us to train individual networks that could identify keypoints across all five camera views and networks that could generalize to novel views—a particularly challenging problem given the small size of hand-labeled datasets typical in keypoint tracking applications.

We experimented with training and testing U-Nets using frames from all camera views (“all”), training on four views and testing on the one held out view (“different”), and training and testing using frames from the same camera view (“same”) (Fig. 4, I and J, and figs. S11 and S12). When training and testing using frames from the same camera view, 450 frames were required to achieve a mean error of 2 mm. More frames were required for the other two scenarios: 850 frames using all camera views and 17,250 frames when attempting to generalize to a novel view. To achieve submillimeter accuracy, we required 25,550 frames for the same camera view and 85,250 frames using all camera views. When generalizing to a novel view, we were unable to achieve submillimeter accuracy with up to 100,000 frames (Fig. 4K). Relative to the discrepancy between human labelers, depending on the body part, our method outperforms human labelers by up to 1.5- to 5.2-fold (fig. S10).

### QD800 can be conjugated to antibodies for targeting specific proteins

**Fig. 5.**
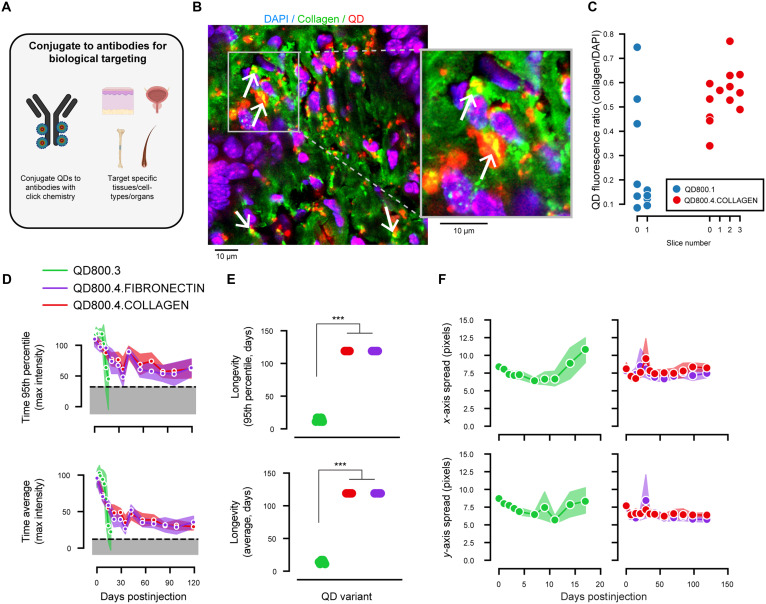
Targeting specific tissues with QD800.4 for substantially increased longevity. (**A**) Strategy for conjugating QD800 to antibodies to target specific tissues, cell types, and molecules. (**B**) Left: Example confocal image from QD800.4.COLLAGEN injection into the back. Tissue was stained with DAPI (for nuclei) and collagen to assess localization. Right: Zoom-in of the region of interest (ROI) indicated by the bounding box in the left image. (**C**) Ratio of QD fluorescence colocalized with DAPI to collagen (*n* = 13 fields of view from *n* = 1 tissue sample/injection for QD800.4 and *n* = 10 fields of view from *n* = 1 tissue sample/injection for QD800.1). (**D**) Fluorescence plotted against days postinjection with same plotting convention as Figs. 2F and 3D. (**E**) Quantification of longevity of fluorescence signal per mouse/camera pair using the 95th percentile across time (*P* = 1.5 × 10^−5^, *U* = 0, *f* = 0 for longevity comparing QD800.3 to QD800.4.COLLAGEN, Mann-Whitney *U* test; *P* = 1.5 × 10^−5^, *U* = 0, *f* = 0 comparing QD800.3 to QD800.4.FIBRONECTIN; *n* = 15 mouse/camera pairs for QD800.3, *n* = 10 mouse/camera pairs for each QD800.4 variant, and *n* = 10 vehicle-injected mouse/camera pairs) and using the average across time (*P* = 1.3 × 10^−5^, *U* = 0, *f* = 0 for longevity comparing QD800.3 to QD800.4.COLLAGEN, Mann-Whitney *U* test; *P* = 1.3 × 10^−5^, *U* = 0, *f* = 0 comparing QD800.3 to QD800.4.FIBRONECTIN). Note that longevity for QD800.4 was as long as they were imaged. (**F**) Spread of fluorescence in *x* and *y* axes for QD800.3 (left) and QD800.4 (right) over time, assessed using the spatial autocorrelation (see Materials and Methods).

Having established QD800.3’s enhanced performance in labeling adipose tissue, we speculated that QDs could also be deployed in applications that required additional biological specificity; that is, QDs could be used in applications that required highlighting specific proteins, cell types, or tissues. As a proof of principle, we adapted a protocol for conjugating QDs to antibodies via click chemistry (see Materials and Methods). Here, we tested directly conjugating QDs to antibodies that target ultralong-lived proteins in the extracellular matrix in skin and joints: collagen and fibronectin (QD800.4.COLLAGEN and QD800.4.FIBRONECTIN; Fig. 5, A to C).

To visualize the dynamics of these QDs in the skin, as an example, we performed QD800.4.COLLAGEN injections along the back of an additional cohort of animals and harvested the tissue for histology (*n* = 2 tissue samples/injections, *n* = 1 with QD800.1 as a control, and *n* = 1 with QD800.4.COLLAGEN; see Materials and Methods). Using immunofluorescence (IF), we stained the tissue sections with collagen I antibodies and a cell nucleus marker and used confocal microscopy to simultaneously visualize QD-related fluorescence and collagen expression–related fluorescence in multiple fields of view (Fig. 5B). The images revealed that QD800.4.COLLAGEN was more likely to colocalize with collagen I relative to QD800.1. To quantify this, we computed the ratio of QD fluorescence in collagen I–rich regions to cell nuclei. We found that QD800.4.COLLAGEN had a median ratio of 0.56 and QD800.1 had a median ratio of 0.15 (Fig. 5C).

We found that both QD800.4 variants outperformed QD800.3, with resolvable fluorescence up to 119 days postinjection in all animals (Fig. 5, D and E). Moreover, we looked at the fluorescence spread of both and compared it to QD800.3 and found that QD800.4’s shape remains stable for as long as we were able to image it in vivo (Fig. 5F).

### QD800 can directly track joint kinematics

Last, we speculated that, since QD800.3 yielded bright, long-lasting discrete points of fluorescence under the skin, we could introduce QD800.3 directly into the knee joint and still acquire a resolvable signal. This would confirm that our method can provide direct visualization of key body parts for precisely quantifying kinematics in mice.

**Fig. 6.**
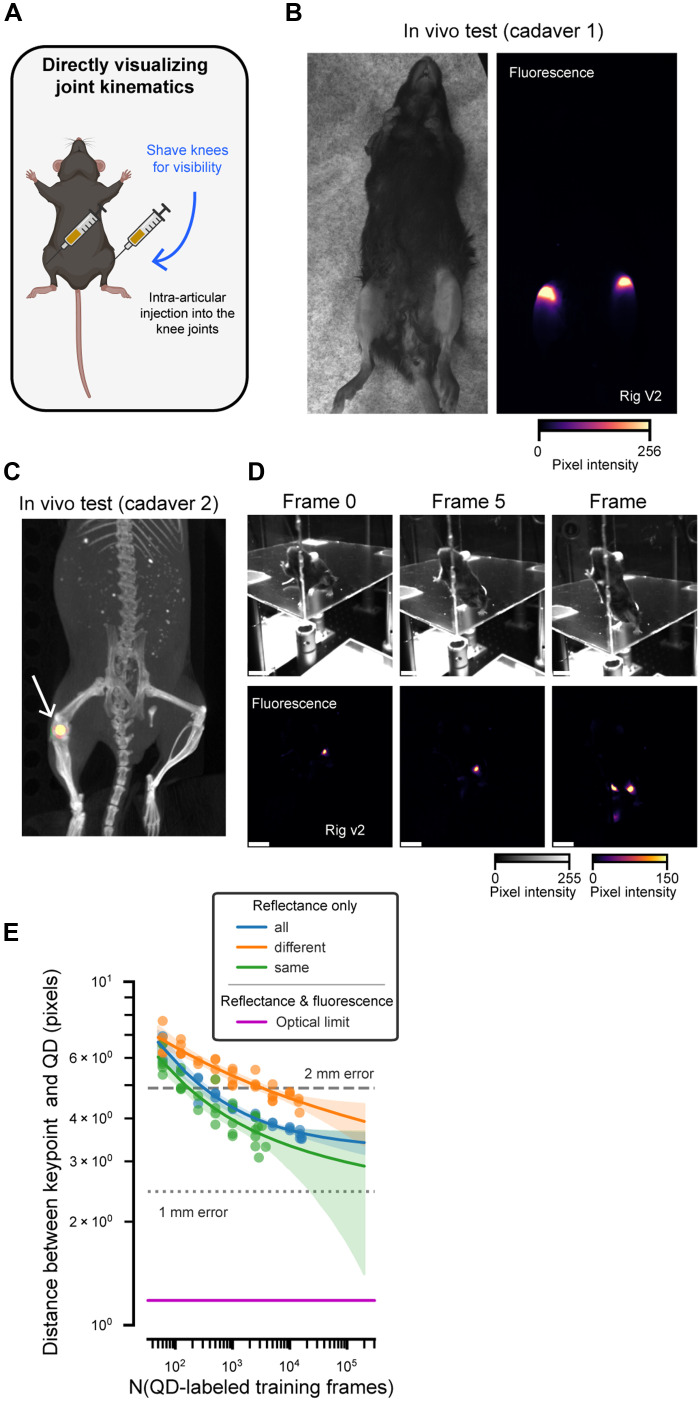
Direct visualization of the knee joint in vivo through intra-articular injection of QD800.3. (**A**) QD800.3 was injected directly into the knee joints after shaving fur. (**B**) In vivo cadaver validation of intra-articular targeting. (**C**) MicroCT image from the right knee joint of mouse cadaver. Fluorescence is overlaid (yellow spot) on top of a reconstructed mouse skeleton. Arrow indicates location of fluorescence spot. (**D**) Frames from video recorded 1 hour postinjection into live mice. Scale bars, 50 pixels. (**E**) Using the same procedure as Fig. 4J, we trained keypoint models to track knee joint location based on surface features. Plotting conventions are the same as Fig. 4J.

Guided by an existing protocol (*49*), we altered our QD injection protocol to allow for intra-articular injection of QD800.3 into both knee joints in a mouse (Fig. 6A). The first test was performed on a mouse cadaver (Fig. 6B). With each QD injection, we confirm that movement of the overlaying skin did not affect the fluorescent spot using a custom-built NIR-camera setup (see Materials and Methods). We further confirmed the location of the injection in relation to the skeleton with a micro–computed tomography (microCT) image from a mouse cadaver using In Vivo Imaging System (IVIS) Spectrum CT (PerkinElmer/Revvity; Fig. 6C and movie S2; see Materials and Methods). In addition, we performed intra-articular injections into the knee joints of live mice and subsequently imaged them in our plexiglass arena (1-hour postinjection; Fig. 6D). We confirmed that the intra-articular injection sites could be reliably tracked as the mice freely moved.

Given the high SNR of QD800.3 directly injected into the knee joints, we hypothesized that a markerless keypoint tracker could be trained to accurately track the position of the joints. To test this, we again hand-labeled a small number of frames (*n* = 872 frames from *n* = 1 mouse) to map each fluorescent point to the corresponding body part (left or right knee joint). These labels were then used to train a U-Net to leverage reflectance and fluorescence data to label the full dataset. Last, we used this dataset to benchmark the ability of the trained U-Net to track the position of the knee joints given surface features (Fig. 6E). Here, we found that 2-mm accuracy could be achieved using 250 frames for the same camera view, 350 frames for all camera views, and 3350 frames when generalizing to a novel view. The number of frames required for 1-mm accuracy exceeds the maximum number of frames that we tested (*n* = 17,901 frames).

To confirm the targeting efficiency of our knee injections, we repeated them in a second cohort of animals following the same protocol (*n* = 4 mice; see Materials and Methods). Seven of eight knee injections were successful on the first attempt, while one knee injection required a second attempt due to glass pipette breakage.

## DISCUSSION

Here, we developed a method called QD-Pi for optically tracking movement in freely moving mice. We show that QDs can be injected into joints and used to directly image joint kinematics. This is a key step toward measuring skeletal movement in animals, and it distinguishes QD-Pi from markerless keypoint tracking methods, which are limited to tracking points on the surface of an animal.

To establish QDs as optical tags, we injected NIR-I–emitting QDs (QD800), free or immobilized on microbeads, in discrete spots in the adipose tissue just beneath the dermis of each animal. We found that the microbead-immobilized particles formed long-lived deposits that can be used as fiducials for tracking keypoints inside the body using standard machine vision cameras. We also found that two commercially available variants were suitable for in vivo use: QDs originally designed for tracking the vasculature (QD800.1) and QDs tailored for cell labeling (QD800.2). However, the fluorescence of both variants decayed to noise levels within 1 to 5 days after injection. This motivated us to design a custom third variant where biotinylated QDs are attached to porous, streptavidin-functionalized agarose microbeads (QD800.3). QD800.3 led to robust labeling with fluorescence that remained resolvable for more than 2 weeks after injection. We then developed a variant (QD800.4) that can be customized for biological targeting, which we used to target QDs to long-lived proteins in the extracellular matrix. Signal from QD800.4 remained resolvable for as long we imaged it, up to 4 months postinjection, a timescale readily compatible with longitudinal systems neuroscience experiments. We also characterize QD localization in vivo and show that the QD800.2 signal is predominantly intracellular compared to QD800.1, which is mostly deposited outside the cell. In addition, our analysis shows that QD800.4.COLLAGEN is more likely to colocalize with collagen I compared to QD800.1. Furthermore, we demonstrated that QDs can be resolved in vivo using substantially different excitation setups. Longer wavelengths may be more desirable in some experimental setups (e.g., to minimize interface with other imaging equipment), while shorter wavelengths enable higher fluorescence levels. Last, we show that QDs can be used to directly visualize joints, a key step in accurately resolving kinematics in mice.

In this study, we tested QD variants in freely moving mice in an open arena. This presents additional challenges in quantification due to variation in excitation power as mice change positions relative to the excitation LEDs. Nevertheless, our method of quantification computes fluorescence as mice occupy different positions in the arena, across five different camera views, and across multiple mice. In addition, we show that QDs can work with substantially different hardware configurations (Rig v1 compared to Rig v2), indicating that our method is robust to variation in excitation and emission optics; this means that downstream users of QD-Pi can tailor optical hardware to their particular needs.

QDs represent an attractive alternative to other tags used in marker-based methods that have been used in rodents. Elegant work from Butler *et al.* (*50*) demonstrated that ultraviolet (UV) ink can be applied to the fur and used to measure a single point in freely moving mice [see also (*26*) for a similar method using nonfluorescent paint applied to rodents]. However, UV excitation light may be visible to the animal (*48*), the ink can fade, and similar markings have been shown to be relatively imprecise (*17*). Since the ink is applied to the outer layer of the skin and fur, it can also be groomed or licked off by the mouse. Other studies have used piercings with highly reflective markers that can be tracked using commercial motion capture systems originally designed for use in humans (*17*, *51*). However, these markers are bulky and still represent motions on the surface of the skin. Another marker-based work from Moukarzel *et al.* applied tattoo ink directly to the tissue surrounding the knee joint of rats (*52*). Although this method targets the musculoskeletal system, it appears to have relatively low SNR since the tattoo ink is simply dark under IR illumination rather than fluorescent (we note that the SNR was not quantified), the ink likely disperses widely around the joint preventing precise localization, and it has only been shown to mark a single joint at a time.

As an alternative to marker-based methods or x-rays, markerless keypoint trackers have capitalized on recent developments in image-based deep learning methods, resulting in a surge of interest in movement tracking (*5*–*10*). However, they often require laborious hand-labeling of training data, which leads to imprecise predictions due to interlabeler jitter and ambiguity in labeling certain keypoints (*14*, *17*). Our method also requires hand-labeling but only of an initial dataset to train a markerless keypoint tracker to identify fluorescence spots. This enabled the creation of a large dataset with “ground-truth” labels for keypoint trackers—that is, labels with well-defined physical positions inside the body—which we call QD-Pi-120K. Using the resulting dataset, we found that only hundreds of frames were required to achieve an acceptable threshold of 2-mm average error with markerless keypoint trackers, in line with prior work (*10*, *50*). However, human kinematic studies typically track kinematics with submillimeter precision, and humans are on the order of 20 times larger than laboratory mice; thus, the relative error of kinematic tracking in mice is still much higher relative to studies of movement in humans. Achieving submillimeter average error or less in our hands requires at least 25,550 labeled frames, with at least 85,250 required if one wants a single network that works across multiple views, or more than 100,000 to generalize to a novel view. Datasets of this size are not feasible with manual labeling, which emphasizes the critical need for techniques like ours that enable the collection of large, high-quality datasets for training the next generation of markerless keypoint trackers. Moreover, in addition to training markerless keypoint trackers, QDs can be used directly for high-resolution kinematic tracking without these steep requirements on the amount of training data.

Despite the impressive performance and ease of use of markerless keypoint trackers, these methods fundamentally rely on surface features of the animal. Prior x-ray videography of freely moving rodents has shown that, even if one can optimally track keypoints on the skin, the skin can distort the movement of the underlying joint (*22*). While we established QD optical tags in the adipose tissue, we also demonstrate intra-articular QD injections. Direct joint tracking via QD-Pi is a promising approach to accurately measure the part of the body under more direct control of motor circuits—the muscles, joints, and bone; this will allow us to more comprehensively map the relationship between neural activity and movement.

However, while using QDs as optical tags is an attractive approach, there are limitations. Signals from QD800.1 and QD800.2 rapidly faded with a timescale of 1 and 5 days, respectively. Prior work has speculated that QD800.2 may quickly leak from cells either through excretion or cell division (*53*, *54*), which could explain our histology results, where we noted that some QD800.2, although less than QD800.1, was deposited outside cells. That said, signals from QD800.4 versions that targeted long-lived extracellular matrix proteins lasted up to 4 months. Using QD800.4.COLLAGEN as an example, we show that QD-conjugated antibodies may colocalize with low-turnover proteins of interest. It is important to note that the efficacy and reliability of such QD-conjugated antibodies are heavily dependent on the binding specificity, affinity, and permeability of a given antibody to bind its intended target.

In addition, resolving signals from QD800 injections in the fatty tissue along the back required shaving the fur. This is subject to the same limitations faced by other agents used in noninvasive biomedical imaging (*55*–*59*), as either nude mice are needed or fur is removed at the imaging region. Moreover, the resolution of our signals also suffered from crowding in certain body parts. While we were able to further analyze and partially account for the spread of our fluorescent points (fig. S9), since our QD solutions are injected by hand, experimental control over particle spread is limited. This may be addressed in future work by performing slow, controlled injections via a syringe pump or other automated microinjection system. This would also provide more precise control over injection volume, which should allow for precise control over fluorescence spot size to prevent signal crowding. Another future direction is to explore more sophisticated peak-finding algorithms, including mean shift or mixture of Gaussian-based clustering.

Another limitation is that the mechanism of QD clearance from the body remains an open question, including for the microbead-immobilized QDs presented here. It has been reported that QDs that were administered intravenously and intradermally are deposited in the liver, lymphatic, and renal systems of mice and rats (*45*, *60*, *61*). Additional investigation into this question may provide insight into further improvements in half-life and signal longevity. While we achieved months-long longevity with QD800.4 variants, we believe that lifelong labeling may be possible given the photostability of QDs.

We note that the dataset generated by our system, QD-Pi-120K, is only an initial step to generating high-quality keypoint tracking datasets at scale. In the future, we plan to further improve the SNR and time resolution of our system. The speed of our current multiplexed imaging system, approximately 30 frames per second, with 17.5 ms between the reflectance and fluorescence exposures. Thus, there is the possibility of minor displacements between the fluorescence data and surface features in QD-Pi-120K during periods of rapid motion. While we were able to successfully reduce this delay to 6 ms without substantial loss of SNR (fig. S13), subsequent versions of the imaging rig will focus on further reductions in time between these exposures to minimize the possibility of this occurring and to increase the effective frame rate. As the imaging speed of our system and SNR is further optimized, in the future, we plan on generating additional benchmark datasets that improve on QD-Pi-120K. Furthermore, with future improvements, we plan on directly characterizing the performance of QD-Pi for capturing body kinematics during ethologically relevant behaviors in mice. This includes locomotion (*62*), grooming (*4*), and paw trajectories (*63*).

Additional future directions include injecting QDs directly into the muscle, bone, and specific tissues within a joint, such as the tendons. Recent developments of fluorescent dyes in NIR-I (*55*) and shortwave infrared (SWIR; 1000 to 2000 nm) (*56*–*58*) range have made it possible to visualize deeper tissue structures, like the skeleton or organs, and perform multiplex imaging in both anesthetized and awake mice through intravenous injections. While we chose to use QDs in the NIR range to facilitate the adoption of our technique, future studies should look at incorporating QDs in the SWIR range, as it may have higher SNR, especially for deep tissue injections (*59*).

Another direction is to label other tissues like the whiskers and potentially internal organs. While we plan to systemically characterize which of our QD variants works best for targeting various body parts, highly specific targeting to cell types, tissues, and molecules will be enabled by further variations on QD800.4. Moreover, QDs have near-ideal optical properties for spectral multiplexing. QDs are available at a wide variety of emission wavelengths spanning most of the visible spectrum into mid-IR with high quantum yields (*64*, *65*), with conveniently overlapping excitation spectra. Consequently, one could easily label separate mice or separate parts of the body with distinct colors.

Last, the markerless keypoint trackers used here are relatively simple models based on the U-Net architecture (*11*). There are now much more complex architectures, e.g., transformer networks (*66*), that could better capitalize on the large datasets that can be produced by our system. In addition, we did not systematically explore bottom-up or top-down architectures, each of which could lead to substantial performance gains.

Thus, we present a method for directly tracking positions inside an animal’s body. In addition to advancing the measurement of kinematics in freely moving mice, we envision that our method can be used to augment markerless keypoint trackers, which have become the standard method for movement tracking in laboratory animals (*5*–*10*). By imaging both the fluorescence from specific points in the body and the surface features surrounding those fluorescent points simultaneously, we have amassed a large dataset that can be used to benchmark models for tracking keypoints in 2D (*5*, *7*, *9*, *10*) and 3D (*6*, *8*). Moreover, in addition to acting as a method for directly measuring the motion of positions inside a mouse’s body, our method will be instrumental in building ground-truth datasets at the scale necessary for constructing foundation models for tracking motion in laboratory animals. Ultimately, this method can be used to resolve complex motor patterns in laboratory organisms with newfound accuracy and precision.

## MATERIALS AND METHODS

We used QD solutions that were suitable for in vivo use. For this manuscript, we used four variants of QDs, all of which have a CdSe core and ZnS shell and are additionally coated with PEG for enhanced biocompatibility (QD800.2 is functionalized with polyarginine peptide instead of PEG).

### QD800 variant 1 (QD800.1)

Because of their prior use in in vivo tracing of the vasculature in mice, the first variant we tried was QTracker 800 Vascular Labels (Thermo Fisher Scientific, #Q21071MP). These were used undiluted (2 μM).

### QD800 variant 2 (QD800.2)

Because of their prior use in in vivo tracking inside of cells, the second variant we tried was the QTracker 800 Cell Labeling Kit (Thermo Fisher Scientific, #Q25071MP). Qtracker nanocrystals (component A, 2 μM) was diluted by half with Qtracker carrier (component B).

### QD800 variant 3 (QD800.3)

Both QD800.1 and QD800.2 were relatively short-lived in the skin. Larger particles, e.g., polymethyl-methacrylate beads, can persist in the skin for up to 5 years (*46*). Hence, we hypothesized that attaching the QDs to larger biocompatible particles would enhance longevity. We found using agarose beads to be ideal due to their porous structure and biocompatibility. Moreover, we used the streptavidin-biotin reaction to immobilize the QDs onto the agarose beads.

High-capacity streptavidin agarose beads (Amid Biosciences, #SA-101-1) and custom-made biotin-conjugated QDs (Thermo Fisher Scientific, per manufacturer’s specification, ≥0.5 mg/ml) were used for agarose bead injections (see fig. S5A for all microbead brand names that were tested). For suspension, sodium alginate (Sigma-Aldrich, #W201502-SAMPLE) was hydrolyzed in 1× phosphate-buffered saline (PBS) to make a 2% (w/v) solution. The beads were spun down, and the supernatant was removed. QD solution was added at 2:1 (v:v) beads-to-QD ratio and gently mixed with the beads by pipetting up and down. The mixture was left to incubate at 40°C for 1 hour and was mixed halfway through the incubation period to ensure saturation. After the incubation, the solution was mixed again and then washed three times with 1× PBS. The supernatant was removed, and the beads were resuspended in 2% sodium alginate in a 1:1 beads-to-alginate ratio to allow for an even distribution during injection.

### QD800 variant 4 (QD800.4)

To enable biological targeting of QDs, we used antibody-based targeting of QD800 to long-lived extracellular matrix–associated proteins, such as collagen and fibronectin. The exact concentration of QDs used in this kit was not disclosed by the manufacturer.

To conjugate QD800 to anti–collagen I + III (Abcam, #ab34710) and anti-fibronectin (Abcam, #ab2413) rabbit polyclonal antibodies, we capitalized on an enzyme- and click chemistry-mediated site-specific antibody conjugation approach (*67*) and used an antibody labeling kit (Invitrogen, SiteClick Antibody Labeling Kit, #S10455). Anti–collagen I + III-QD800 and anti–fibronectin-QD800 antibody conjugates are referred to as QD800.4.COLLAGEN and QD800.4.FIBRONECTIN, respectively. Briefly, the antibody was concentrated using membrane filtration. Next, the carbohydrate domain of the antibody was modified for azide attachment using uridine-5′-diphosphate-*N*-azidoacetylgalactosamine labeling. Azide-modified antibody was then concentrated using membrane filtration and conjugated to dibenzocyclooctyne-labeled QD800. To enhance the yield and concentration of conjugated antibodies (QD800.4) and to filter out unconjugated antibodies, we purified QD800-conjugated antibodies using membrane filtration.

### Animals and surgical procedure

All procedures were approved by the Georgia Institute of Technology Institutional Animal Care and Use Committee (protocol #A100557). Thirty-three male and 17 female (7 to 20 weeks old) C57BL/6J mice were purchased from the Jackson Laboratory and kept under a reverse 12-hour light/12-hour dark cycle with ad libitum access to food and water. Specifically, for control, *n* = 3 mice were not injected with any material (blank), and *n* = 5 were injected with vehicle (vehicle, *n* = 3 were used for Rig v1 and *n* = 2 for Rig v2. Blank and vehicle were combined in the control group). For QD800 injections, *n* = 3 were injected with QD800.1, *n* = 3 with QD800.2, *n* = 6 with QD800.3 (*n* = 3 were used for Rig v1 and *n* = 3 for Rig v2), and *n* = 5 were injected with QD800.3 intra-articularly into the knee joints (*n* = 3 were injected for in vivo recordings and *n* = 2 cadavers were used for imaging). For QD antibody injections, *n* = 2 were injected with vehicle (described as *n* = 2 for Rig v2 above), *n* = 2 were injected with QD800.4.FIBRONECTIN, and another *n* = 2 were injected with QD800.4.COLLAGEN. For IF experiments, *n* = 4 were injected with QD800.1 (tissues from *n* = 2 were harvested at +30 min, and *n* = 2 were harvested at +4 hours postinjection), *n* = 2 were injected with QD800.2 (tissues were harvested at +4 hours postinjection), and n = 2 were injected with QD800.4.COLLAGEN (tissues were harvested at +8 days postinjection). For exposure time and volume experiments, *n* = 9 were injected with QD800.2. For knee injection success rate experiments, *n* = 4 were injected with QD800.3 intra-articularly into the knee joints. For Fig. 2B, *n* = 1 mouse was injected with QD800.2 (sex was not determined for this animal). The animals were anesthetized with 4% isoflurane mixed in air using a nose cone. The procedures were carried out under 1.8 to 2% isoflurane anesthesia on a heating pad and completed within 30 min. The fur along the spine was shaved and further treated with a hair removal cream. The QD mixtures were injected using a sterile pulled glass micropipette (Drummond 3-000-210-G) created using a Sutter P-2000 laser puller (parameters: heat, 450; filament, 4; velocity, 150; delay, 175; pull, 35). The micropipette was attached to a modified positive-displacement microinjector (Drummond 3-000-510-X). Specifically, the stainless steel plunger of the microinjector was cut to enable the pulled micropipette to be safely attached to the microinjector. The tip of the micropipette was cut to the desired diameter for different QD800 injections (fig. S5G). The QD mixtures were delivered to each of the 14 injection sites subdermally: the paws (dorsal and ventral), the tail (base, midsection, and tip), and the back (upper, middle, and lower midline dorsal region). Following each injection, 70% ethanol and triple antibiotic ointment were applied to minimize potential infections. The animals were allowed to recover in their cage for 1 hour after the procedure.

A volume of 2 μl of QD800.1 and QD800.2 was delivered to the 14 injection sites subdermally with a 0.1- to 0.2-mm-diameter micropipette. For agarose bead injections, the diameter of the micropipette was 0.3 to 0.4 mm to allow for the passage of the beads. The injection volume was changed to 4 μl to maximize the signal and was delivered to the same 14 injection sites subdermally.

An injection volume of 4 μl was also used to deliver QD800.4.FIBRONECTIN and QD800.4.COLLAGEN to the same 14 injection sites subdermally with a 0.1- to 0.2-mm-diameter micropipette. The vehicle was repeated with the same injection volume alongside these experiments. In addition, the fur along the spine was shaved and treated with a hair removal cream under anesthesia 4 and 6 weeks after QD800.4 injections to enhance the keypoint signal from the back of the mice.

The protocol for intra-articular knee injection was based on the protocol from Pitcher *et al.* paper and modified accordingly (*49*). The fur around the legs was shaved and treated with a hair removal cream. A volume of 4 μl of QD800.3 was delivered to each knee joint using a micropipette with a diameter of 0.25 to 0.3 mm to allow for bead passage and accurate access to the joint. For in vivo injections, postoperative analgesia was given at the start of the procedure, and following each injection, 70% ethanol and triple antibiotic ointment were applied. To confirm the targeting of the injections, the animals were placed under NIR illumination and imaged with a machine vision camera outfitted with a long-pass filter (see below for details on imaging hardware). The skin around the knee was tugged, and the signal was observed live to see if the fluorescence spot moved with the skin. Only the signal that remained stationary while moving the skin was deemed intra-articular. The animals were allowed to recover for 1 hour after the procedure. Here, *n* = 3 mice were injected, and two of three mice showed strong fluorescence in only one knee joint. The third mouse showed strong fluorescence in both knee joints and was thus used for analysis in Fig. 6E. To assess the success rate of knee injections, we performed a second round of intra-articular injections into the knee joint. Here, *n* = 4 animals were injected. Seven of eight knee injections were successfully injected on the first try, while one knee was repeated because of pipette breaking.

For IF experiments, the back of the animals were shaved and treated with a hair removal cream. QD800.1, QD800.2, or QD800.4.COLLAGEN was delivered to multiple injection sites on each animal’s back, following the same surgical procedure and respective parameters described above. The back skin tissues from the injection sites were harvested individually, where each tissue corresponds to one injection, at +4 hours for QD800.1 and QD800.2, at +30 min for QD800.1 control, and at +8 days for QD800.4.COLLAGEN postinjection. Extracted tissues for each condition were pooled and stored in individual tubes. For imaging and quantification, we selected *n* = 2 tissues for QD800.1 and *n* = 2 tissues for QD800.2 to compare QD signal enrichment inside versus outside of cells; and *n* = 1 tissue for QD800.1 as a control and *n* = 1 tissue for QD800.4.COLLAGEN to compare their colocalization with collagen I antibody.

### Recording arena

A plexiglass arena was created from clear cast-acrylic panels (McMaster-Carr, #8560K184) to form an open-top cube with 29.845-cm edge length (i.e., same width, depth, and height). Interdigitated patterns were cut into the edges of panels to ease fitting together. Panels were glued together using acrylic plastic cement (Sci-Grip, #10315). Custom 3D-printed molds were secured to the bottom acrylic panel (Torr-Seal Epoxy, Varian), and screw-to-expand brass inserts were inserted into the molds to secure 0.25- to 20-inch (50.8 cm) set screws. The other end of the set screws was inserted into Thorlabs 1-inch (2.54 cm) optical posts to, in turn, secure to an optical breadboard placed on top of a leveled frame (PFM52503).

### Recording sessions

Mice were placed into the plexiglass chamber and imaged for ~3 to 5 min per session.

### Recording hardware—Version 1

The plexiglass arena was filmed using five hardware-synchronized NIR-optimized Basler USB3 cameras (acA2040-90um). For wide field-of-view imaging, the cameras were outfitted with a Thorlabs machine vision lens with an 8-mm focal length (MVL8M1). To prevent imaging of QD excitation light, long-pass (MidOpt LP830-55) and polarization filters (PR1000-55) were secured to the lens. Polarization filters were rotated until excitation light was minimized. For exciting QDs at wavelengths compatible with imaging through skin, we used NIR-I–emitting LED lights (SL246-730IC) outfitted with polarization filters (PA371-S82). For collecting reflectance images, IR-emitting LED lights were used (SL246-850IC). The lights were triggered in a temporally multiplexed configuration so that fluorescence and reflectance data could collected near-simultaneously using the following sequence: (i) IR lights on for 10 ms, (ii) all lights off for 1 ms, (iii) NIR lights on for 23 ms, and (iv) all lights off for 1.5 ms. Power to the LED lights was supplied from a benchtop voltage source (B&K Precision BK1550) (fig. S1B). To test whether shorter exposure times could be used, a faster sequence was used in fig. S13 only: IR lights on for 2 ms, lights off for 1 ms, NIR lights on for 8 ms, and all lights off for 6 ms (here, a larger gap was used to allow for frame readout). Lights were triggered using 5-V signals generated from an Arduino Uno, which was also used to trigger camera exposures via the General Purpose Input/Output (GPIO) lines on the Basler cameras. The illumination sequence was repeated for ~5 min for each recording session. The cameras were arranged in a pentagonal formation surrounding the plexiglass arena to capture mice at multiple angles.

### Recording hardware—Version 2

The following modifications were made to the recording hardware version 1 (see above section) to maximize the SNR. First, to enhance the excitation of the QD800 nanoparticles, we decided to slightly blue-shift the excitation light (Fig. 4A). The 730-nm LED lights were replaced with 660-nm LED lights (Advanced Illumination SL-S100150W-660). Because of the blue-shifted excitation light, polarizing filters were no longer needed to filter out stray light, so they were removed to enhance signal levels. In addition, the long-pass filters were modified to accommodate the new wavelength, so they were replaced with 780-nm long-pass filters (MidOpt LP780-55).

### Recording hardware—Optical limit

The optical limit of our system, presented in Figs. 4 and 6 was estimated by calculating the Nyquist limit given a pixel size of 5.7 μm, an object distance of 304.8 mm (the approximate distance from each camera to the center of the plexiglass arena), and a lens focal length of 8 mm.

### Recording hardware—Setup for visualizing the success of knee injections

We constructed a compact setup under which we could image QD fluorescence from intra-articular knee injections in anesthetized mice. LED lights (660 nm, Thorlabs M660L4 driven by a Thorlabs LEDD1B) outfitted with an adjustable lens (Thorlabs SM1U25-A) were used for exciting QDs. Fluorescence was imaged using an 830-nm long-pass filter (MidOpt LP830-28) mounted to the camera lens. Images were collected either using a Basler a2A3840-45ucBAS or acA2040-90um outfit with a 12-mm focal length lens (Thorlabs #MVL12M23) connected to a laptop running Basler pylon software. Nonfluorescence images were collected simply by removing the long-pass filter.

### Recording software

Camera control and image acquisition software were written in Python. Custom software was also written for the Arduino to trigger the cameras over the appropriate GPIO line, along with the LED lights.

### Analysis of quantum dot fluorescence

To assess the brightness and longevity of QD injections, we computed a simple summary metric for each camera view and each session (Fig. 1F). First, to remove any contribution of the background, a rolling background (1500 frame sliding window, nonoverlapping) was subtracted from the fluorescence frames (fig. S2A). Next, to summarize the intensity on a per-frame basis, we computed the maximum pixel value across *x* and *y* for every frame. Last, to summarize either the average or peak intensity across time for each camera view and each recording session, we computed either the mean or the 95th percentile across maximum frame intensities.

To confirm that full-frame calculations did not introduce artifacts into our analysis, we repeated the calculation using segmentation masks and allowed us to ignore all pixels that did not belong to the mouse (fig. S2). To estimate the segmentation mask on reflectance frames, we manually labeled 1395 frames using the segments.ai platform. Then, we used the nvidia/mit-b3 variant with default settings using pretrained weights from Huggingface.

Spatial autocorrelation was computed using the scipy.signal.fftconvolve function on background-subtracted fluorescence data. To minimize redundant calculations, the spatial autocorrelation was computed on every 200th frame. The autocorrelation on each frame was normalized such that the peak at the center was 1.

### Markerless keypoint tracking (standard keypoints, tail, back, and paws)

To track keypoints, we used SLEAP (*10*). First, reflectance and fluorescence frames from all five camera views were alpha blended (90% fluorescence and 10% reflectance), uploaded to segments.ai, and were hand-labeled (*n* = 862 total frames). Hand-labels were verified by a second labeler. Next, the hand-labeled, blended frames were used to train a single-instance U-Net to automatically identify body parts that coincided with fluorescence peaks. Eight hundred sixty-two frames from all five cameras were hand-labeled using the segments.ai platform. We performed a grid search over key hyperparameters (fig. S12) and used the following: filters 64, filters_rate 2.0 (this was set to 1.5 for sample efficiency experiments in Figs. 4K and 6E for computational efficiency since many models were fit), middle_block true, up_interpolate true, max_stride 64, stem_stride NULL, sigma (for head) 2.5, and output_stride 4.0. The final model had 502,521,418 parameters, and the model for sample efficiency experiments had 27,153,429 parameters. The following augmentation settings were used (any options not listed were set to false): rotate_min_angle −15, rotate_max_angle +15, translate_min −50, translate_max +50, scale_min 0.85, scale_max 1.15, gaussian_noise_mean 5.0, gaussian_noise_stddev 1.0, contrast_min_gamma 0.5, contrast_max_gamma 2.0, random_flip true, and random_flip_horizontal true. This network was then applied to our entire dataset. The predicted *x*/*y* position of each body part was then corrected using the fluorescence channel. To precisely localize each keypoint prediction, for each prediction, we computed the fluorescence center within a 10-pixel radius surrounding the predicted keypoint.

To compute the center of fluorescence points, we first used the OpenCV findContours function to identify a group of fluorescent points closest to the predicted keypoint. All pixels that did not belong to this group were set to 0. Next, a bivariate Gaussian was fit to the remaining fluorescence data. The center of the Gaussian was used as an estimate of the center of fluorescence (see fig. S9 for a comparison of methods for estimating the fluorescence center).

The fluorescence center of mass distance from ground-truth labels was compared with raw keypoint predictions in Fig. 4 (G and H). We refer to fluorescence localization as refinement. The center of mass was then used as the ground-truth *x*/*y* position for training and assessing the markerless keypoint trackers shown in Figs. 4K and 6E.

As a rule of thumb, given an ~10 mm span between many key landmarks on a mouse (*18*), we assume that the average error of a given keypoint tracker should not exceed 2 mm, which is approximately 4.9 pixels given our optical setup. A more stringent criterion of submillimeter error is likely required for accurately quantifying rodent kinematics (2.45 pixels on our setup), since submillimeter precision is common in human motion capture, and humans are approximately 20 times larger than mice (*19*–*21*). Here, we define error as the average L2 distance between the network-identified keypoint and the corresponding QD fluorescence peak.

### Filtering keypoint predictions to automatically build new training datasets

To ensure that high-quality keypoints were used to build large training datasets for Figs. 4K and 6E, we postprocessed keypoint predictions using the following rules. First, we assumed that any large jumps in position between frames indicated a tracking error, so keypoints were excluded if they moved more than 30 pixels (L2 distance) between neighboring frames. Next, to filter outliers, we dimensionally reduced the *x* and *y* coordinates of the 10 keypoints (back bottom, back middle, back top, tail base, tail middle, tail tip, and left/right fore/hindpaw) using principal components analysis (PCA). Here, we selected the number of PCs required to drop the cumulative mean squared reconstruction error by 90%. Next, we considered a keypoint visible if its keypoint confidence score exceeded 0.2. Of the visible keypoints, we then set thresholds on the minimum amount of fluorescence and the maximum distance to the nearest QD center for the keypoint to be considered valid; both values scaled with keypoint confidence. Less confident keypoints required higher fluorescence and needed to be closer to the nearest QD center. The minimum fluorescence peak linearly scaled from 75 for predictions with a score of 0.7 to 25 for a score of 1.0. The maximum distance is scaled from 5 for predictions with a score of 0.7 to 15 for a score of 1.0. To prevent inclusion of frames with large difference between the number of “visible” keypoints and the number of “valid” keypoints, we linearly scaled the number of accepted dropped keypoints (visible − valid) from 0 for frames with 3 or fewer keypoints to 3 for frames with 10 keypoints. Last, to exclude frames with outliers, we removed any frames where the distance between any pair of keypoints exceeded 300 pixels.

### Markerless keypoint tracking (knee joints)

For tracking of knee joints, all settings were the same as with standard keypoints, except for the following modifications. Eight hundred seventy-two fused fluorescence and reflectance frames were manually labeled. Then, since the fluorescence data were extremely sparse (one or two fluorescence spots per frame), we trained a markerless keypoint tracker to identify keypoints using only the reflectance images. All postprocessing thresholds were the same, except that the low confidence score used to linearly scale the fluorescence peak and distance to fluorescence center thresholds was set to 0.5 (rather than 0.7). Also, since there were only two keypoints when labeling the knee joints, the PCA postprocessing filter was skipped.

### Estimating fluorescence spread with autocorrelation

To estimate the spread of fluorescence across time (Fig. 5F), we took the spatial autocorrelation from every 200th frame (see above for more details) and fit a bivariate Gaussian distribution using scipy.optimize.least_squares. The SDs along the *x* and *y* axes were used as estimates of spread.

### Human keypoint annotation and analysis

Manual keypoint labeling was carried out independently by four labelers for reflectance data and six labelers for overlaid reflectance and fluorescence data. Each labeler was presented with a series of images containing either overlaid reflectance and fluorescence camera frames (*n* = 862 frames) or reflectance frames only (*n* = 271 for the standard set of 10 keypoints shown in Fig. 4, and a separate *n* = 274 frames were labeled for knee joints shown in Fig. 6) and asked to label keypoints visible on the camera frame. The frames were sampled randomly across all five cameras. Labelers all agreed on where keypoint locations should be, on average, before labeling, and individual frames were labeled independently. For fluorescence data, labelers marked the center of the fluorescent dot as the keypoint for the relevant body part. For reflectance data only, labelers estimated the location of keypoints based on the reflectance image used as a reference. If an area was not visible due to the current angle, the area was labeled as “hidden.” Labelers were blinded to the identity of the mice they were labeling.

### Estimating keypoint performance scaling with the number of frames

To estimate how keypoint localization error (Figs. 4K and 6E and fig. S12D) scaled with the size of the training dataset, we fit a power-law decay function of the form

$$y=\frac{a}{{\left(x+1\right)}^{b}}+c$$

where *x* is the number of training frames and *y* is the keypoint localization error. The parameters *a*, *b*, and *c* were fit using the scipy.optimize.least_squares module. Then, to estimate how other performance characteristics (fig. S12E) scaled with training dataset size, we used an inverted form of the same function

$$y=a\times \left(1-\frac{1}{{\left(x+1\right)}^{b}}\right)+c$$

which was fit using the same method.

### Camera calibration and estimation of QD-Pi error by position in arena

Intrinsics and extrinsics for all five cameras were estimated using a plexiglass cube with four separate ChArUco boards taped onto the outer faces—the top was left open to access the inside of the cube, and the bottom face was used for mounting onto the optical breadboard. The cube was spun by hand, and frames were synchronously captured. Intrinsics and extrinsics were estimated using routines from OpenCV. Subsequent bundle adjustment of extrinsics was performed using the scipy.optimize.least_squares function.

To estimate the precision of QD-Pi as a function of position in the arena (fig. S11), we then used the keypoints predicted by the “reflect + fluo” model shown in Fig. 4 (E to H). To produce an unbiased estimate of prediction error, no quality control thresholds were used (see the “Filtering keypoint predictions to automatically build new training datasets” section). Then, to resolve keypoints in 3D, keypoints were triangulated using standard multiview triangulation (*68*). Triangulated points and extrinsics were used to compute the distance of each keypoint relative to each camera view and its angular distance from each camera’s optical axis. We use the distance between the predicted keypoint and the nearest QD fluorescence center to estimate the error in the prediction. The distance from the camera was used to convert the error from pixels to mms.

### Histology

For QD800 histology, tissues of interest at the injection sites (back skin, paws, and tail) were harvested and kept in 4% paraformaldehyde solution for 48 to 72 hours at 4°C before transferring them to 1× PBS solution. Before cryopreserving with sucrose, the paws and the tail were further dissected to collect the skin at the injection site. The tissues were placed in 15% and then 30% sucrose in 1× PBS until they sank. The tissues were embedded in optimal cutting temperature compound (Sakura Finetek, #4583) and frozen with dry ice. They were then cryosectioned at 50 μm using a cryostat (Thermo Fisher Scientific, CryoStar NX70), collected with microscopy slides, and stored at −80°C until further processing. The sections were thawed, washed twice with 1× PBS, incubated in 4′,6-diamidino-2-phenylindole (DAPI; 1 μg/ml in water) (Thermo Fisher Scientific, #D1306) for 3 to 5 min, and washed thrice with 1× PBS. The slides were then coverslipped using an antifade mounting medium (VECTASHIELD, #H-1700).

The back skin tissues that were collected for IF assay and wheat germ agglutinin (WGA) staining went through the same tissue processing steps as described above. However, before freezing with dry ice, all samples except QD800.1- and QD800.2-injected samples went through vacuum infiltration. They were then cryosectioned at 20 μm, transferred onto frosted glass slides, and stored at −20°C until further processing.

### Immunofluorescence and WGA staining

To visualize the cell membrane, we performed WGA staining. For this, the sections were thawed, washed thrice with 1× PBS, incubated in WGA conjugated to Alexa Fluor 488 (10 μg/ml in 1× PBS) (Thermo Fisher Scientific, #W11261) for 1 hour, and washed four times with 1× PBS. The slides were then coverslipped using antifade mounting medium with DAPI (VECTASHIELD, #H-1800).

For collagen I antibody IF staining, we used a primary antibody derived from a species other than rabbit (host species of antibody used in QD800.4.COLLAGEN) or mouse to prevent antibody cross-talk and cross-reactivity. The sections were first thawed and washed thrice with 0.1 M phosphate buffer (PB). Then, the sections were blocked with 10% horse serum (Thermo Fisher Scientific, #16050114) in 0.1 M PB solution containing 0.3% Triton X (PBTx) for 30 min at room temperature. Sections were then incubated in PBTx solution containing 1% horse serum and goat anti–type I collagen antibody (1:500; SouthernBiotech, #1310-01) overnight at room temperature. Sections were then washed four times with 0.1 M PB solution and further incubated in PBTx solution containing donkey anti-goat antibody conjugated to CF488A (Sigma-Aldrich, #SAB4600032) for 1.5 hours at room temperature. They were washed four times with 0.1 M PB solution and coverslipped using antifade mounting medium with DAPI (VECTASHIELD, #H-1800).

### Imaging of immunofluorescence and staining

For QD800 histology, brightfield or fluorescence images were acquired at ×4 and ×10 magnification using a Nikon Eclipse Ti2 widefield epifluorescence microscope equipped with a penta-band excitation filter cube set (Semrock, LED-DA/FI/TR/Cy5/Cy7-A-000), Lumencor Spectra III light engine, and a Hamamatsu Orca Flash 3.0 complementary metal-oxide semiconductor sensor. The following excitation filters were used: DAPI (excitation wavelength or λex = 365 nm) and Cy7 (λex = 730 nm). Imaging parameters (e.g., exposure, laser power, etc.) for each excitation filter were kept consistent across all image acquisitions.

For IF and colocalization studies, images were acquired at ×20 and ×63 magnification using a laser scanning confocal microscope (ZEISS, LSM 900). The following excitation/emission filters were used: DAPI (λex = 353 nm, emission wavelength or λem = 465 nm), Alexa Fluor 488 (λex = 493 nm, λem = 517 nm), QD800 (λex = 300 nm, λem = 799 nm). Imaging parameters (e.g., laser power, binning, pixel dwell time, etc.) for each excitation/emission filter set were kept consistent across all image acquisitions.

### Quantification of immunofluorescence and staining

To estimate the tendency for QD800.2 to enter cells (Fig. 2, D and E), we calculated the ratio of QD fluorescence inside of cells to outside of cells. To achieve this, we took our confocal images of DAPI, WGA, and QD fluorescence and used the DAPI and WGA channels to estimate the regions of interest (ROIs) for each cell using Cellpose (*69*). Here, the flow threshold was set to 0.4, and the cellprob_threshold was set to 0.0. Then, the average QD fluorescence was computed per ROI along with the average QD fluorescence outside of all ROIs. The ratio between these two quantities then provided an estimate of the tendency for QDs to enter cells.

Second, we used confocal images of DAPI, collagen I, and QD fluorescence (Fig. 5, B and C) to establish whether QD800.4.COLLAGEN colocalized with collagen. We thresholded collagen-related fluorescence using the Otsu’s method to establish a collagen mask. DAPI fluorescence was also thresholded using the same technique. Then, we calculated the ratio of QD fluorescence that colocalized with the collagen mask to QD fluorescence that colocalized with DAPI.

### IVIS spectrum CT imaging

Intra-articular knee injection of QD800.3 was performed into both knees of a mouse cadaver. The bottom half of the mouse was shaved and further treated with hair removal cream to reduce imaging artifacts.

IVIS Spectrum CT Imaging system (PerkinElmer/Revvity) was used to image the right knee in fluorescence tomography mode using a set of trans-illumination points combined with CT-based reconstruction of the skeletal structure. Living Image 4.7.4 software was used to reconstruct the fluorescence light source (QD800) based on this trans-illumination at selected locations. The reconstructed fluorescence was then overlaid with both surface reconstruction of the animal and its skeletal structure to create a 3D representation of the injected QD800.3. Only results from the right knee are shown since the left knee imaging parameters were not appropriately optimized for this demonstration.

### Statistics

All hypothesis tests were two-tailed and nonparametric. Where appropriate, we list the exact *P* value, sample size (along with definition of a sample), and the exact value of the relevant test statistic. Results are expressed as mean ± SD. In all figures, *P < 0.05, **P < 0.01, and ***P < 0.001. Effect sizes for Mann-Whitney *U* tests are given as common language effect sizes, *f*. Effect sizes for Wilcoxon signed-rank tests are given as the rank-biserial correlation, *r*. Boxplots conventions are as follows. Boxes extend from the first quartile to the third quartile of the data, with a line specifying the median. The whiskers extend to the farthest datapoint within 1.5-fold of the interquartile range of the data. Outliers beyond these points were not plotted.
