# Supplementary material for: High-resolution in vivo kinematic tracking with customized injectable fluorescent nanoparticles
Source: Sci Adv. 2025 Oct 1;11(40):eadu9136. doi: 10.1126/sciadv.adu9136 (PMC12487877; doi:10.1126/sciadv.adu9136)
Supplement: Supplementary file 1 — Figs. S1 to S13 Legends for movies S1 and S2 [file sciadv.adu9136_sm.pdf]

Science Advances
AAAS

Supplementary Materials for

High-resolution in vivo kinematic tracking with customized injectable fluorescent nanoparticles

Emine Zeynep Ulutas *et al.*

Corresponding author: Jeffrey E. Markowitz, jeffrey.markowitz@bme.gatech.edu

Sci. Adv. **11**, eadu9136 (2025)
DOI: 10.1126/sciadv.adu9136

The PDF file includes:

Figs. S1 to S13
Legends for movies S1 and S2

Other Supplementary Material for this manuscript includes the following:

Movies S1 and S2

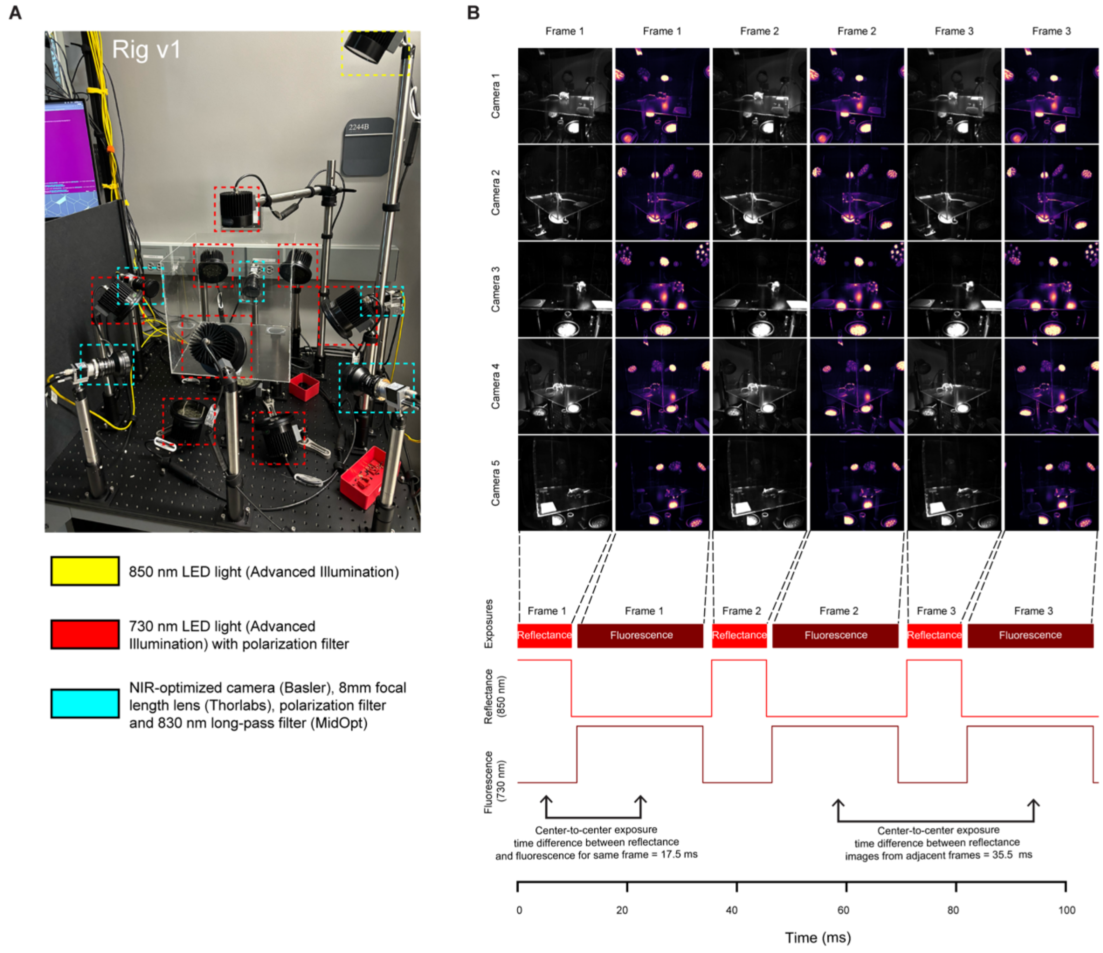

Fig. S1. QD imaging rig version 1. (**A**) Shown is the rig and plexiglass arena used for imaging quantum dots (QDs) in freely moving mice. All data included in **Figs. 1-3** and **Figs. S2-4** were collected using this setup. (**B**) Schematic of the illumination sequence used for temporal-division multiplexing. Note that the fluorescence images presented here are not background-subtracted.

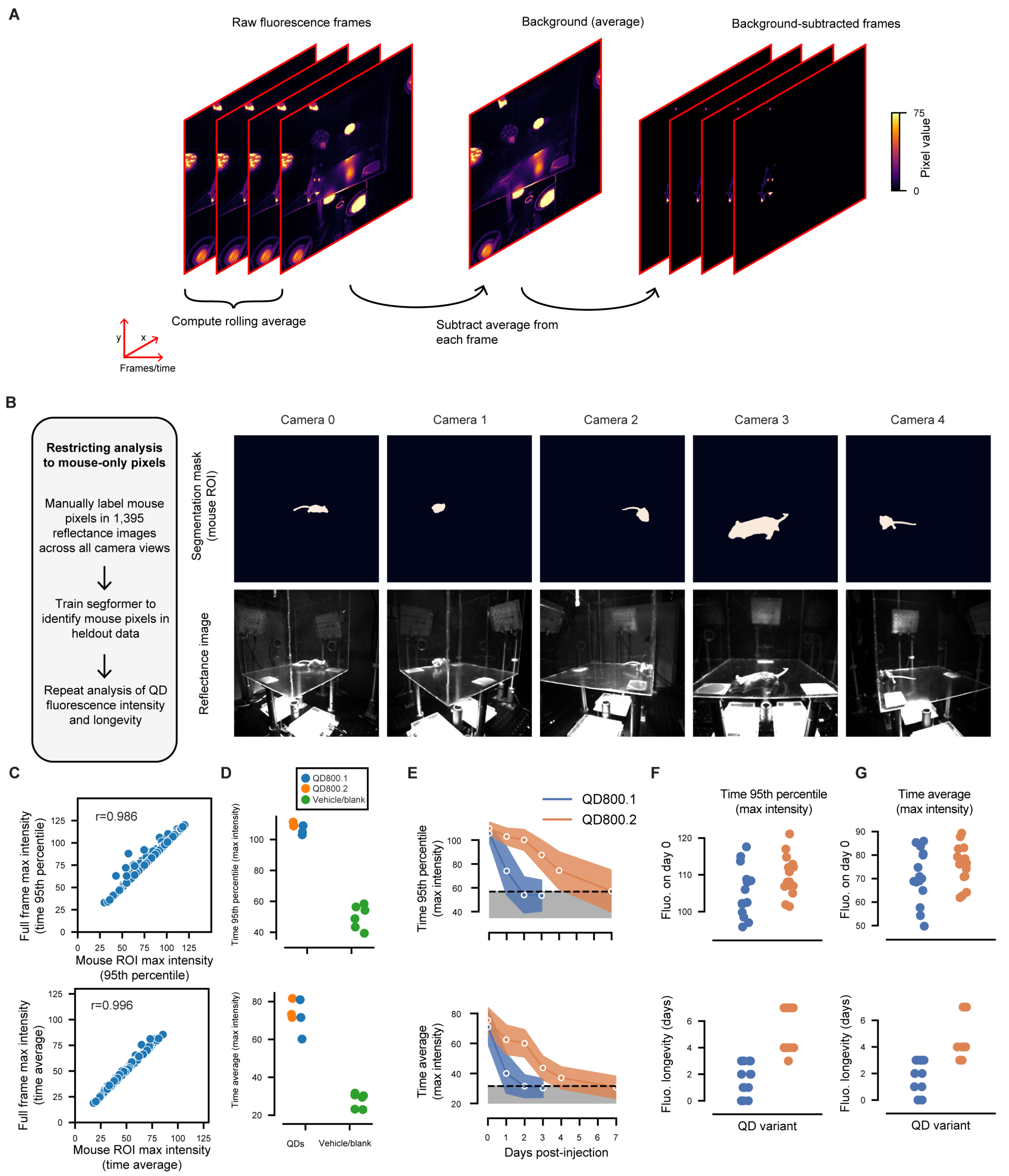

Fig. S2. Fluorescence data pre-processing. (**A**) To remove static elements from the scene (*e.g.*, excitation LEDs), the background of the image was computed using a non-overlapping 1500-frame-long sliding window. All visualization and quantification of fluorescence data uses background-subtracted data. (**B**) Quantification of fluorescence intensity and longevity in **Figs. 1-5** used peak fluorescence across the entire frame and subsequently computed either the 95th percentile or the average across frames. To verify that non-mouse pixels did not contribute meaningfully to these estimates, we manually labeled 1,395 reflectance images from all five

cameras to specify which pixels were occupied by the mouse. Then, we trained a Segformer using the nvidia/mit-b3 variant with pretrained weights from Huggingface. Example segmentation masks and the corresponding reflectance images are shown. (**C**) Either average or 95th percentile of max intensity computed using either the full frame or the mouse ROI. (**D**) Analysis in **Fig. 1G-H** repeated using the peak fluorescence computed with the mouse ROI rather than the full frame. (**E-G**) Analysis in **Fig. 2F-H** repeated using the peak fluorescence computed with the mouse ROI rather than the full frame.

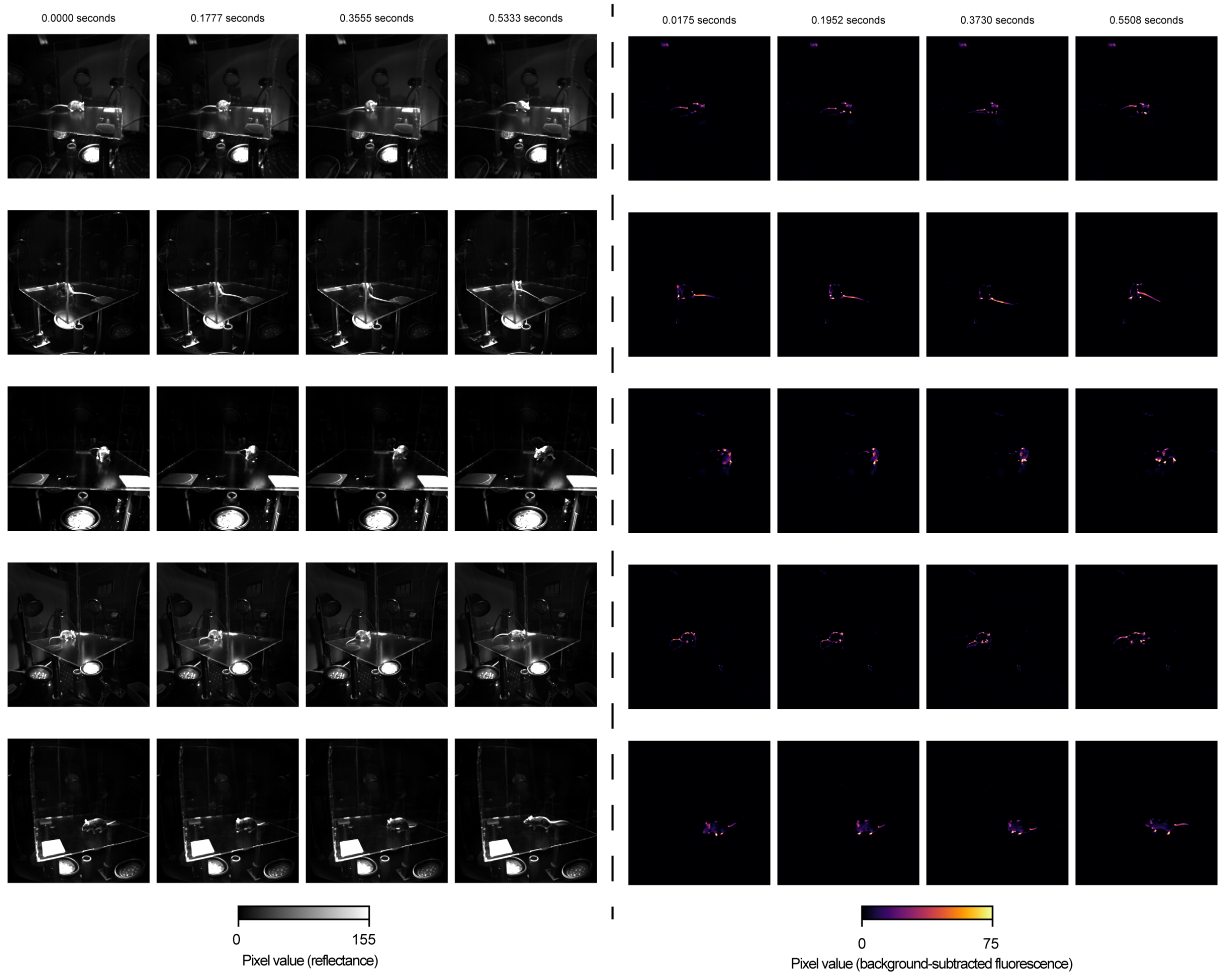

Fig. S3. Example reflectance and fluorescence data from a single session. Shown are reflectance (left) and fluorescence data (right) for four frames—timestamps are given at the top—from all five hardware-synchronized cameras. Data from a mouse injected with QD800.2.

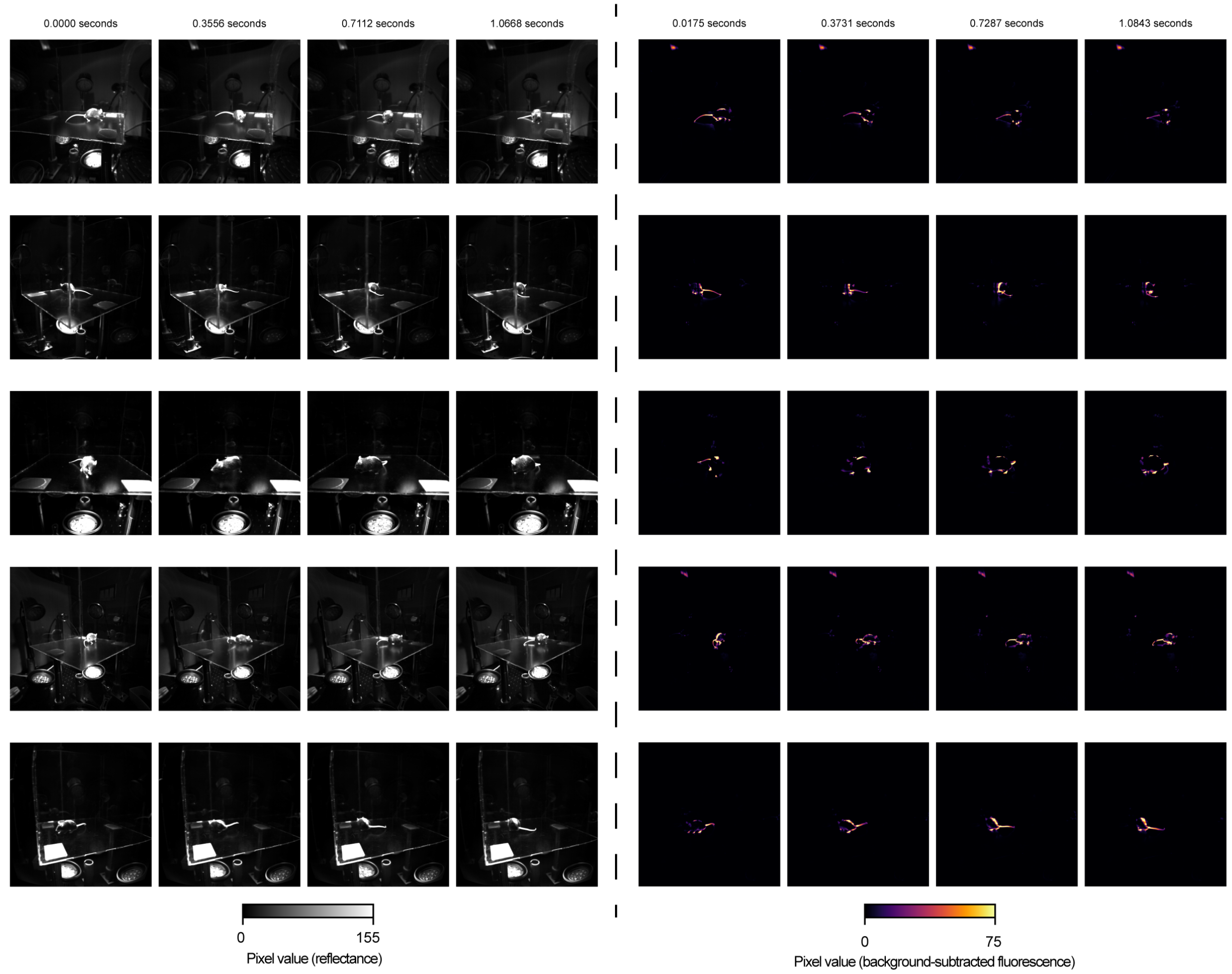

Fig. S4. Example reflectance and fluorescence data from a single session. Same layout as **Fig. S3**, except a different session is shown. Data from a mouse injected with QD800.2. The mouse shown here is different from the mouse shown in **Fig. S3**.

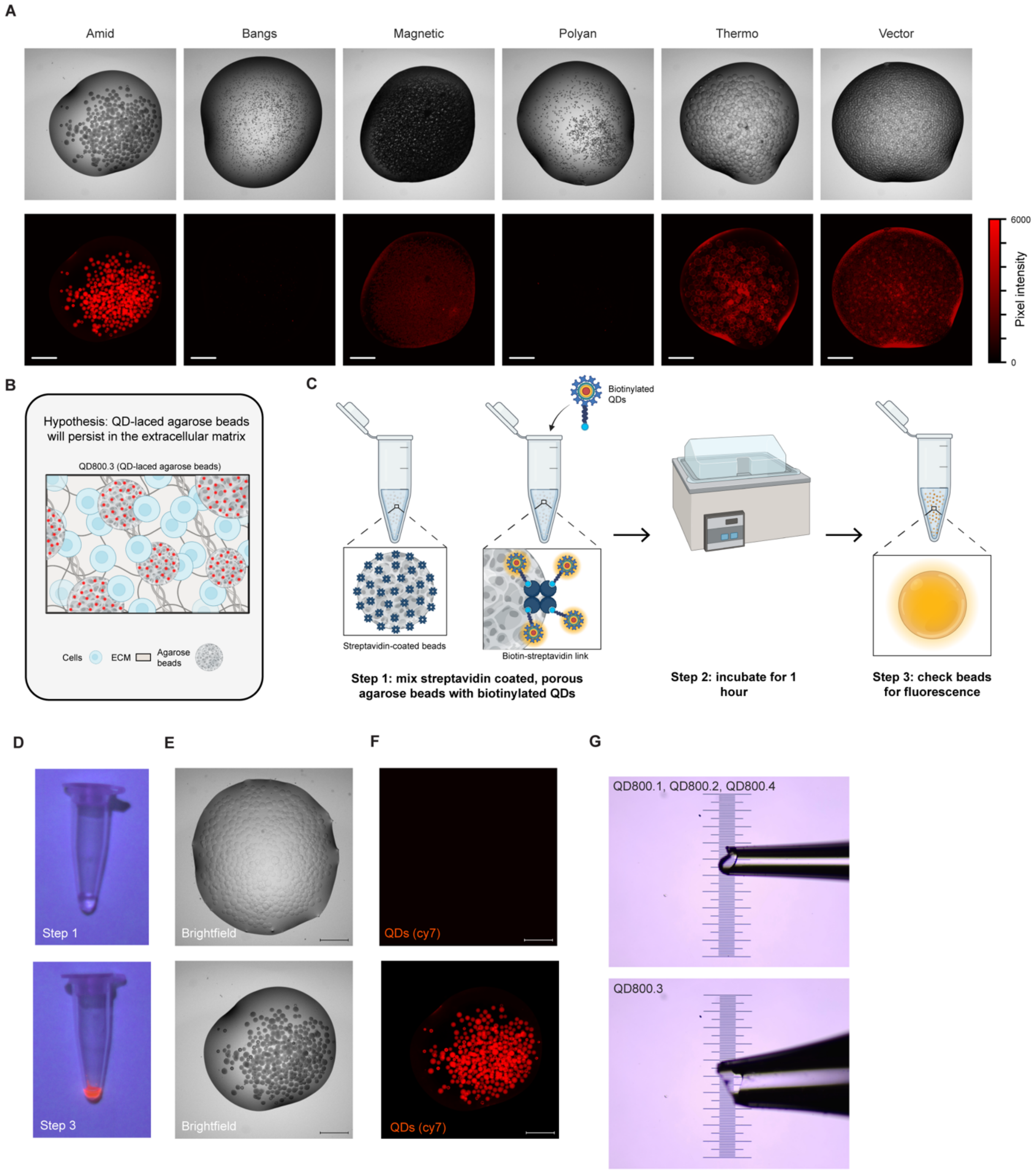

Fig. S5. Extending the longevity of QD fluorescence by attaching to microbeads. (**A**) A droplet from six different microbead brands (Amid Biosciences, #SA-101-1; Bangs Laboratories, #CP01008; Resyn Biosciences, #MR-STM002; PolyAn, #105-21-020; Thermo Scientific, #20357; Vector Laboratories, #N-1000-002) mixed with biotinylated QDs imaged under brightfield (top) and fluorescence (bottom) using Cy7 excitation/emission (see Methods for details). Scale bar represents 500 µm. (**B**) Schematic of our hypothesis—attaching QDs to a relatively large, biocompatible, porous agarose beads will lead to stabilization of QD

fluorescence. We call this QD800.3. (**C**) Protocol for QD800.3. Streptavidin-coated agarose beads are mixed with biotinylated QDs and incubated for 1 hour at 40ºC, mixed halfway through the incubation period. The supernatant is removed, the solution is washed 3 times with 1X PBS, and the beads are resuspended in 2% sodium alginate. (**D**) Images of agarose beads (top) and our agarose bead QD mixture (bottom). Images were taken under 405 nm illumination. (**E**) A droplet of agarose beads (top) or the agarose bead QD mixture (bottom) under brightfield illumination. Scale bar represents 500 µm. (**F**) Fluorescence from the same droplet using Cy7 excitation/emission (see Methods for details). Scale bar represents 500 µm. (**G**) Pipette tips for QD injections. Each minor division is 10 µm.

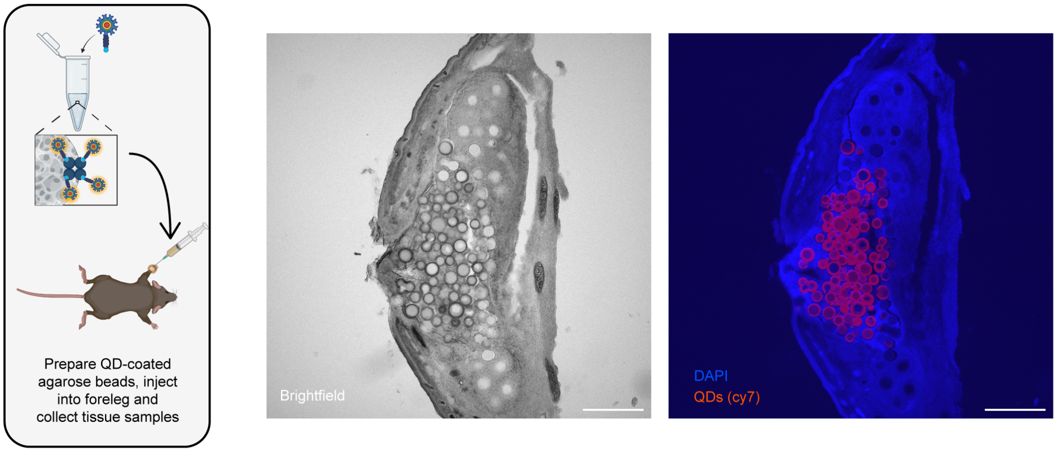

Fig. S6. Histology from a left forepaw injection of QD800.3 (agarose bead QD mixture). Left, schematic of experiment. QD800.3 was injected into the left forepaw, then tissue was harvested 1-week post-injection and imaged. Middle, brightfield image of injection site. Right, fluorescence image of the injection site. Scale bar represents 500 µm.

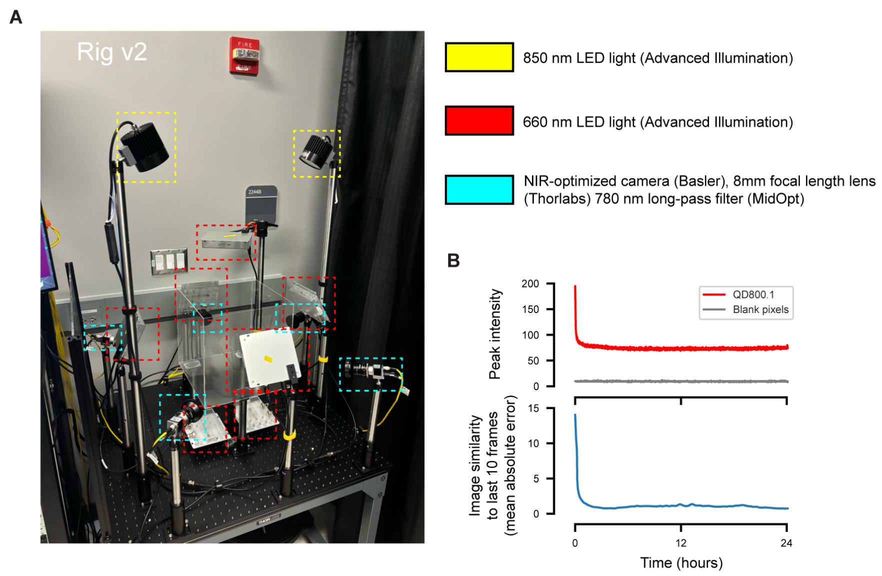

Fig. S7. QD imaging rig version 2. (**A**) All data included in **Figs. 4-6**, **Movie S1**, and **Figs. S8-S13** was collected using this setup. (**B**) QDs were pipetted onto a slide and imaged in the plexiglass arena once every 30 seconds for 24 hours to measure photostability in rig version 2. Excitation light was left on continuously. Top, an ROI was selected around the slide, and the peak was computed over the ROI per frame. Shown is the peak intensity in the image ROI and the peak intensity of a blank patch of the same frames. Bottom, similarity of the ROI to an average of the last ten frames. Due to drying, the QD droplet rapidly changes shape over the first hour. Once the spatial distribution of fluorescence is stable, the QDs are highly photostable.

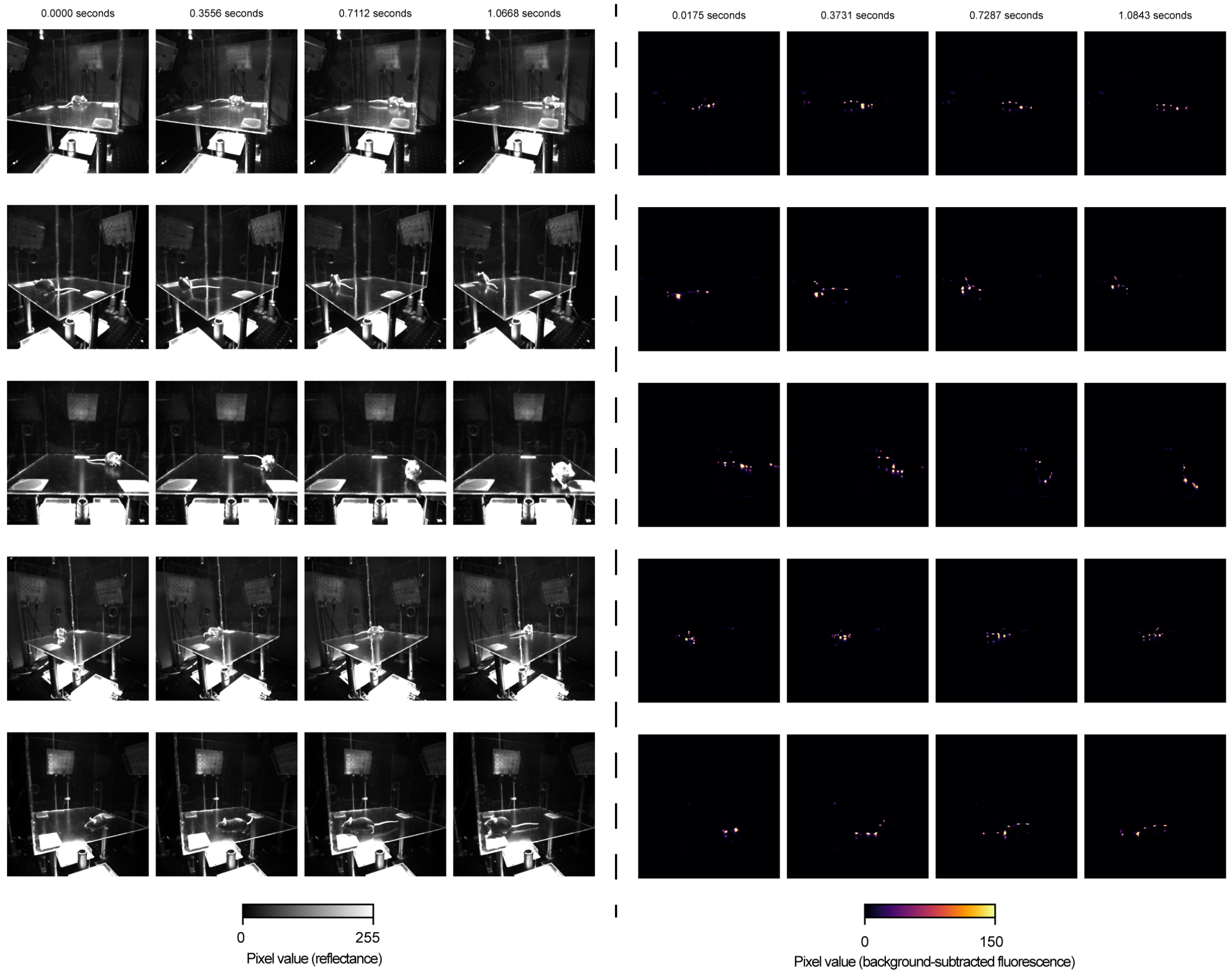

Fig. S8. Example reflectance and fluorescence data from a single session collected using rig version 2. Same layout as **Figs. S3, S4**. The mouse shown here was injected with QD800.3.

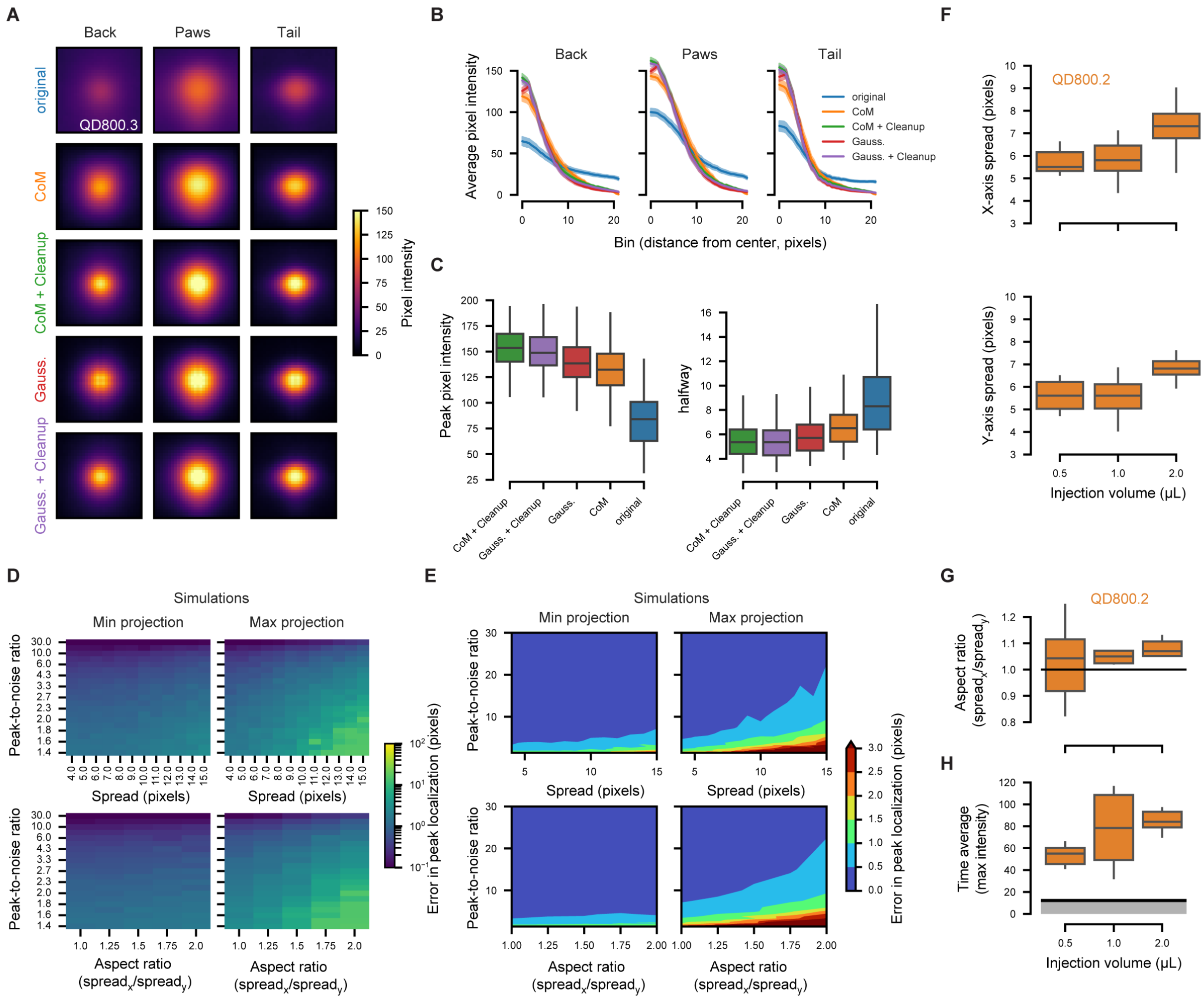

Fig. S9. The spatial profile of QD fluorescence and its impact on marker localization. (**A**) Using keypoint predictions from the "reflect+fluo" model used in **Fig. 4E-G,** we took a 30 x 30-pixel window around each predicted keypoint location, and averaged windows across all predictions for each body part. For computational efficiency, we randomly sampled 2,000 windows for each keypoint. Here, "Back" combines the bottom, middle, and top back keypoints; "Paw" combines all paw keypoints, and "Tail" combines the base, middle, and tip of tail keypoints. Each row shows the fluorescence after using a different method for refining the keypoint prediction using fluorescence: original, no fluorescence refinement; CoM, using only the center of mass; CoM + cleanup, a contour was fit to the fluorescence close to the predicted keypoint, pixels not inside of the contour were masked out prior to computing the center of mass; Gauss., fitting a bivariate Gaussian to the fluorescence data and using the mean to detect the center; Gauss. + cleaning, using a contour fit same as CoM + cleanup and then fitting a Gaussian to the pixels after applying the cleanup mask. (**B**) Histogram of the intensity data shown in (**A**) using radial bins. (**C**) Quantification of the data shown in (**A**). Left, boxplot of the peak pixel intensity at the center of the cropped window. Right, distance from the center to reach 50% of peak intensity. (**D**) To assess the impact of fluorescence intensity and the spread of fluorescence on detection of the center of fluorescence, we performed simulations where we varied intensity,

spread of fluorescence, and spread aspect ratio (ratio of spread along the x-axis to spread along the y-axis). We varied each of these three parameters and performed 100 simulations per parameter setting. In each simulation, we used our algorithm for detection the center of fluorescence (Gauss. + cleanup from (**A**)) and calculated how far the detected center was from the true center. Here, we show performance across all three parameters. Min projection indicates that, for the parameter not shown in the heatmap, we compute the minimum error. Max projection indicates that, for the parameter not shown in the heatmap, we compute the maximum error. (**E**) Filled contour plot of the simulations shown in (**D**). (**F**) To determine the effect of payload volume on fluorescence parameters, we repeated injections of QD800.2 at three volumes (2.0 μL is the original volume used for **Figs. 1-3**). Here we show boxplots of the spread of fluorescence along the x-axis and y-axis, quantified by calculating the standard deviation along the x-and y-axes from a Gaussian fit to the spatial autocorrelation profile (n=2 mice per injection volume viewed from n=5 cameras, so n=10 camera/view pairs per injection). (**G**) Boxplots of aspect ratio per injection volume. (**H**) Boxplots of time average of peak fluorescence intensity per injection volume. Gray region indicates 99th percentile from vehicle injected mice (same mice as in **Fig. 5D**).

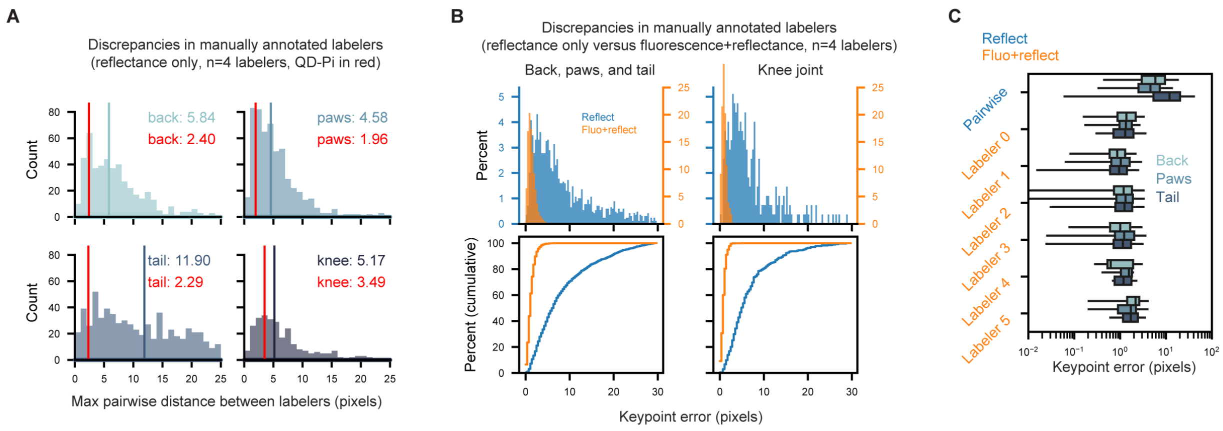

Fig. S10. Assessing the accuracy of manual annotation with and without quantum dot fluorescence. (**A**) Histograms of maximum pairwise distance between n=4 labelers who each annotated the same n=271 reflectance frames with the 10 keypoints shown in **Fig. 4D**, and an additional n=274 reflectance frames with the 2 knee joints. Shown is the maximum pairwise distance across labelers per keypoint/frame pairs for the back keypoints, paw keypoints, tail keypoints, and knee joints. Vertical lines and associated numbers are the median of each distribution. In red is the median of the top 5 SLEAP models trained to predict keypoints from all camera views ("all" from **Fig. 4K** or **Fig. 6E**). (**B**) Top, histograms of discrepancies across labelers for reflectance frames (blue), and the discrepancy between labelers and QD fluorescence centroids with frames with alpha-blended fluorescence (orange). Bottom, cumulative histograms of the same data shown on top. (**C**) Boxplots of pairwise errors across labelers for reflectance frames along with the discrepancy between labelers and QD fluorescence centroids for each of 6 different labelers.

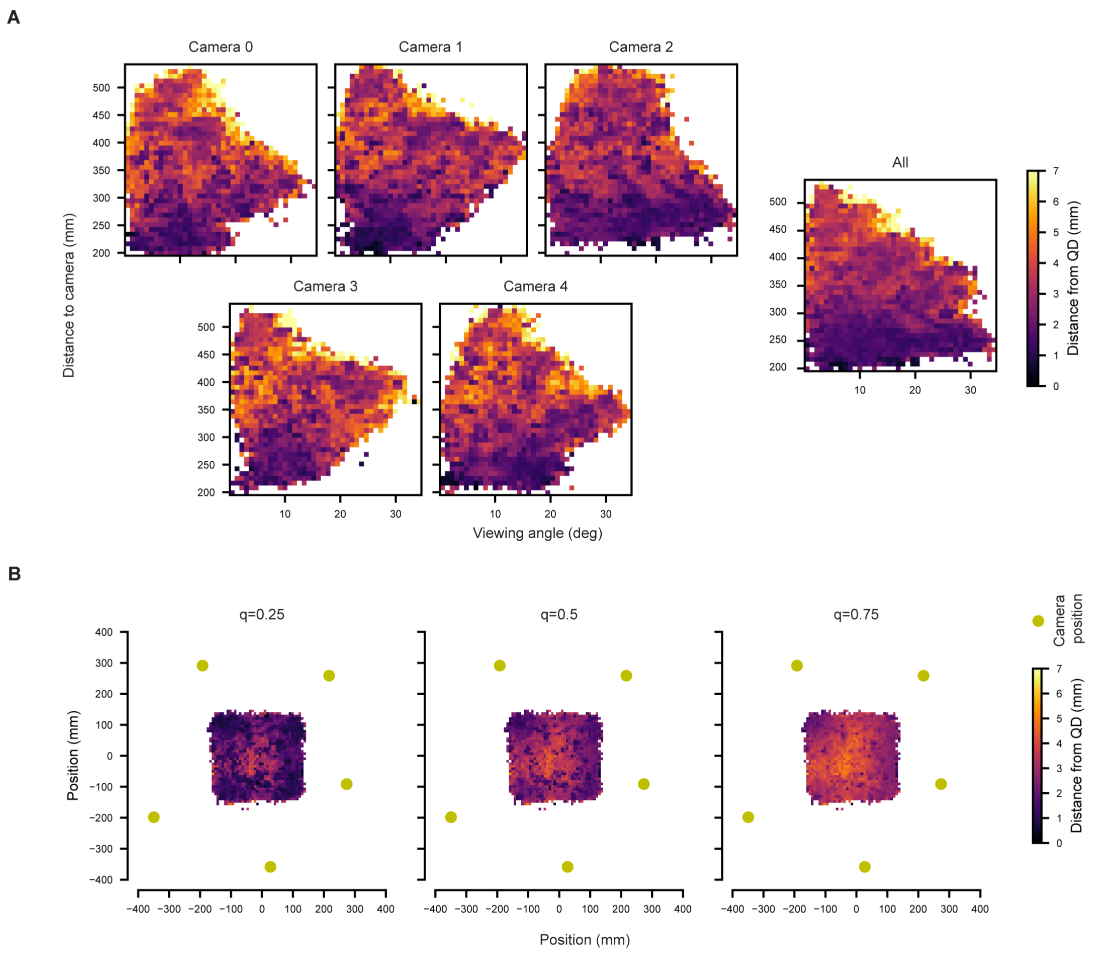

Fig. S11. Assessing the accuracy of reflectance and fluorescence-based keypoint detection at different positions in the arena. Our 5-camera system was calibrated using a set of 4 ChArUco boards arranged into a cube. The cube was manually spun while we collected reflectance exposures. Next, OpenCV was used to estimate the intrinsics and extrinsics of each camera. Bundle adjustment was performed to optimize extrinsics with respect to reprojection error using standard technique (see **Methods**). Then, using the model that predicted keypoints using both fluorescence and reflectance data from **Fig. 4E-G**, we triangulated fluorescence-refined keypoints to estimate the x,y,z position of the mouse and then calculated the distance between predicted keypoints and fluorescence centroids as a function of position in the arena. (**A**) Left, median distance between predicted keypoints and fluorescence centroids shown as a function of distance from each camera and the viewing angle. Right, median distance computed across all cameras. (**B**) Distance between keypoints and QD fluorescence centroid shown as a function of position in the arena. First, for each keypoint, we calculated the error (distance between predicted keypoint and fluorescence centroid) for each camera. Next, we kept the error from the camera that was closest to the centroid of the mouse. Finally, we show the 0.25, 0.5, and 0.75 quantiles of the distribution of errors at each position in the arena.

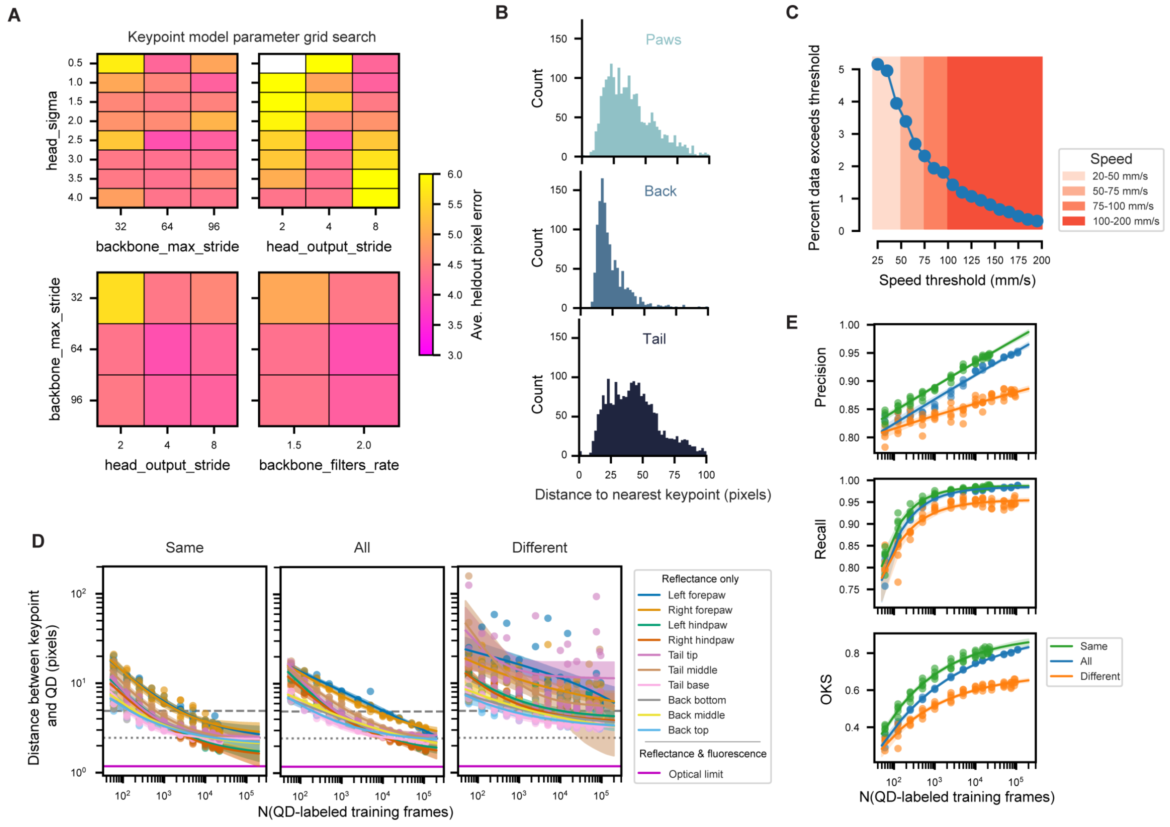

Fig. S12. Details of QD-Pi-120K and models used in Fig. 4. (**A**) For the "reflect+fluo" model from **Fig. 4E**, we determined the best set of parameters through a grid search of key U-Net parameters. Shown is the average heldout pixel error for each parameter set. For the parameters not shown in each heatmap, we computed the minimum error across all settings. (**B**) For the dataset used to train models in **Fig. 4K** (QD-Pi-120k), we computed, for each keypoint, the distance to the nearest neighbor. (**C**) To determine the percent of QD-Pi-120k frames in which the mouse is moving at various speeds, we calculated the velocity of the mouse's centroid using triangulated keypoints (see **Fig. S11** and **Methods** for details) various thresholds between 25-200 mm/s and calculated the percent frames above each threshold. (**D**) Performance of the models shown in **Fig. 4K** broken down by body part for each condition. Conventions are the same as **Fig. 4K**. (**E**) Different performance metrics for the models shown in **Fig. 4K**: precision, recall, and object keypoint similarity (OKS). Line reflects the median inverted power-law decay fit, and the shaded region indicates 95% bootstrap confidence interval.

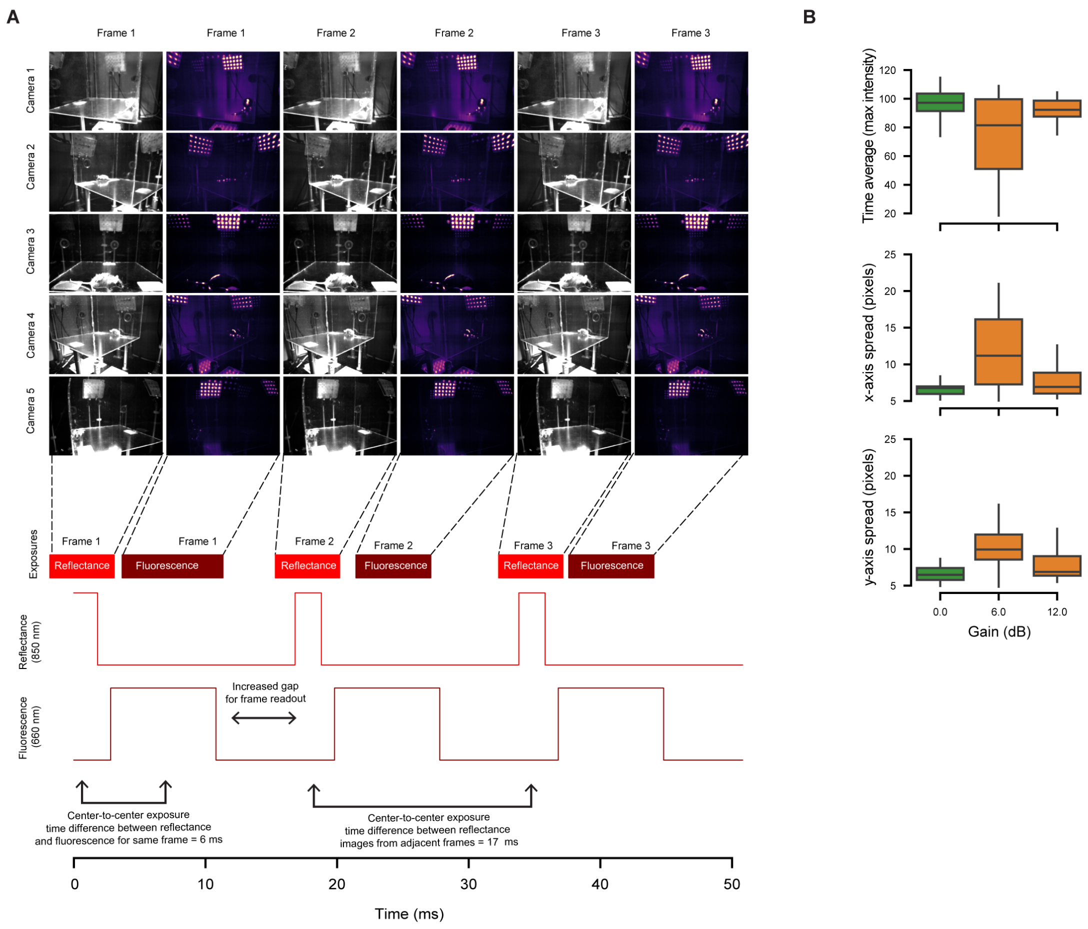

Fig. S13. Increasing the speed of version 2 of the imaging rig through reduced exposure times and increased analog gain. (**A**) Schematic of the illumination sequence with shorter exposure times. Note that here fluorescence images are not background subtracted. (**B**) Top, time average of peak fluorescence intensity from n=5 mice injected with QD800.2 imaged at different exposure times and with different analog gains on each camera. Middle, spread along the x-axis from mice imaged at different exposure times and gains measured using the standard deviation along the x-axis of the 2D autocorrelation function from the fluorescence channel. Bottom, spread along the y-axis, computed using the same method as for the x-axis.

Movie S1. Example reflectance and fluorescence video from all camera views from a single session. Shown is data from a single session with QD800.3, imaging rig v2.

Movie S2. Series of X-ray images of mouse legs overlayed with fluorescence in transaxial plane. A scroll through the transaxial plane to demonstrate localization of the fluorescent signal with respect to the right knee joint.
